# Remedial applications of biosynthesized metal oxide nanoparticles against pathogenic microorganisms

**DOI:** 10.1186/s11671-026-04955-2

**Published:** 2026-10-02

**Authors:** Mathu Metha K, E Ranjith Kumar, Ranjithkumar Rajamani, Ling Shing Wong, Uun Yanuhar, Sinouvassane Djearamane

**Affiliations:** 1 Department of Physics, KPR Institute of Engineering and Technology, Coimbatore, 641407 Tamilnadu India; 2 Department of Biotechnoloy, Sri Ramakrishna College of Arts & Science, Coimbatore, 641 006 Tamil Nadu India; 3 Faculty of Health and Life Sciences, INTI International University, Persiaran Perdana Bandar Baru Nilai, Putra Nilai, Nilai, 71800 Negeri Sembilan Malaysia; 4 Study Program of Aquatic Resources Management, Faculty of Fisheries and Marine Sciences, Universitas Brawijaya, Malang, East Java Jl. Veteran, Indonesia; 5 Department of Biomedical Science, Faculty of Science, Universiti Tunku Abdul Rahman (UTAR), Kampar Campus, Jalan Universiti, Bandar Barat, Kampar, Perak, 31900 Malaysia

**Keywords:** Metal oxide nanoparticles, Microbes, Antibiotic resistance, Biological synthesis, Antimicrobial activity, Biomolecules, Good health

## Abstract

The biosynthetically prepared metal oxide nanoparticles, which function effectively as antimicrobial agents, are used considerably to lessen the harmful effects posed by microorganisms that have become antibiotic-resistant and have kept spreading ever more on a vast scale in environments. The increasing adversity in the biomedical field necessitates the development of materials that fulfill biomedical requirements, and biosynthesized nanoparticles have received significant attention, as they offer substantial benefits by being biocompatible—the critical parameter in need. This review explores assorted metal oxide nanoparticles like aluminum oxide, bismuth oxide, cadmium oxide, cobalt oxide, copper oxide, iron oxide, magnesium oxide, manganese oxide, nickel oxide, samarium oxide, silver oxide, titanium oxide, tin oxide, and zinc oxide; synthesis methods assisted with biomolecules, including doped metal oxides and their nanocomposites; and it also investigates their physicochemical properties, factors affecting antimicrobial efficacy, and potential to replace conventional antibiotics. The main objective is to provide an in-depth review of biomolecule-assisted metal oxide nanoparticles as effective, sustainable, and promising antimicrobial agents for promoting good health.

## Introduction

### Nanomaterial’s significance

Globally, the recent escalating predicament crisis in public health care, such as pandemics, has expedited the development and implementation of novel solutions [1–6]. Presently, it has been extremely challenging to manage the augmenting disease caused by microbes, termed multidrug-resistant organisms (MDROs) [5–8]. The pathogens that possess resistance to existing antibiotics by evading intended targets, utilizing multiple pathways, and showing resilience against multiple drugs leads to increased morbidity and mortality drives to antimicrobial resistance (AMR) [8–11]. Therefore, there is a critical requirement for unconventional, biocompatible, and meticulously designed antimicrobial drugs [12–16]. This antibiotic resistance effectuated us into the challenging era of antibiotic-resistant pathogens, a grave danger to biodiversity stemming from mismanagement of antibiotics [2, 6, 9]. It has been recorded that since 1990, the mortality rate owing to antibiotic resistance has increased, and the World Health Organization considers microbial resistance a “silent pandemic” [17]. It’s expected that approximately 10 million people will die on account of antimicrobial resistance pathogens, according to the UK government’s report [18, 19]. Infectious diseases spread by microbes are leading to epidemics and threaten the world both in terms of global health and the economy, as they are particularly prevalent among malnourished and less immune-responsive populations in underdeveloped and developing countries. Therefore, these need to be diagnosed and medicated as early as possible [20]. This pernicious issue is skilfully and competently dealt with by metal oxide nanoparticles, endowed with inherent antimicrobial potency synthesised via nanotechnology, a cutting-edge approach [3, 15, 21, 22].

Nanotechnology is characterised by its capability to synthesise, engineer, modify, utilise, characterise, and produce materials with desired properties for specific functions under the nanoscale (1–100 nm), encompassing atomic, molecular, and supramolecular levels [7, 10, 16, 17, 20]. Its transformative realm exists across multiple domains like electronics, energy science, agriculture, optics, space industries, biological sciences, biotechnology, cosmetics, catalysis, biosensors, food, medicine, pharmaceutical sciences, photoelectrochemical applications, the chemical industry, the textile industry, communication, and environmental remediation [23–38]. The integration of nanotechnology has a profound impact on medicine, as it offers substantial effects for treatment, diagnosis, monitoring, control of disorders or diseases, detection, and prevention [1, 3, 7, 16]. Nanomaterials in health care applications play roles as gene delivery agents, antimicrobial agents, antiplasmodial agents, anti-biofilm agents, anticancer agents, antiviral agents, antidiabetic agents, antioxidant agents, drug delivery agents, anti-inflammatory agents, imaging agents, anticoagulants, antiparasitic agents, anthelmintics, and tissue engineering materials due to the versatile attribute of the large surface area of nanoparticles, which promotes interactions with microbial cell membranes [21, 25, 28, 31–33, 39–44]. Bio-derived nanoparticles are pre-owned matter from ancient times in Ayurvedic bhasmas [9, 45]. Also, practitioners have employed them as silver thread, silver salts, and metal salts for suturing, treatment, and cleaning wounds [9, 45]. From these it’s evident that metal oxides and their derivatives have a long history as antimicrobial agents [9]. The distinctiveness of nanoscale materials lies in their small size, tunable morphology, size-dependent properties, abundant active sites, high reactivity, free dangling bonds, quantum effects, exceptional stability, large surface plasmon resonance, superparamagnetism, large surface area-to-mass ratio, and surface-enhanced Raman scattering, which are entirely different from bulk counterparts [3, 15, 16, 21]. Nano-dimensioned materials obey quantum mechanics rather than Newtonian physics, with the least influence from gravitational force and major governance by electromagnetic forces [33, 36, 46–49].

### Nanomaterials classification and synthesis

**Fig. 1:**
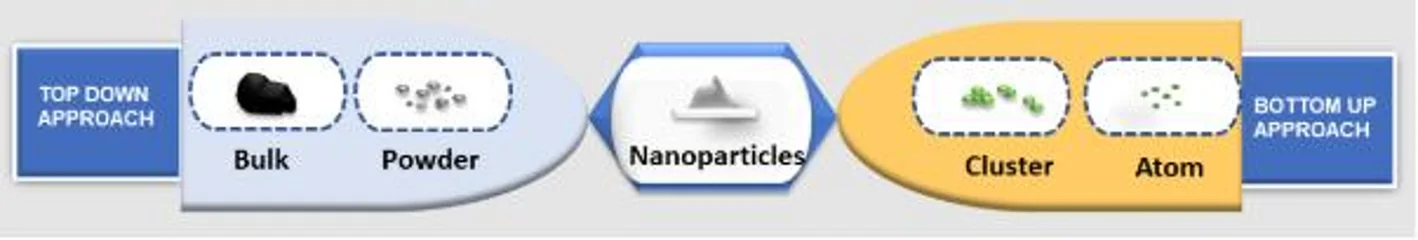
Schematic representation of top down and bottom-up approach involved in the synthesis of nanomaterials

Nanoparticles are classified based on criteria such as their dimensions, principle of synthesis, route of synthesis, composition, and shapes [45]. Among these signifiable classes of classification are those based on chemical nature: organic, which are degradable, non-toxic matter like dendrimers and liposomes; and inorganic, including metals, semiconductors, magnetic and metal oxides, and carbon-based nanoparticles—fullerenes, graphene, carbon nanotubes, and carbon nanofibers [36, 45]. As nanoparticles are capable of exhibiting the peculiar habit of producing desired physicochemical characteristics upon coordination, this enables their use in numerous applications [9, 13, 35]. It is essential to monitor their size, crystallinity, porosity, roughness, and shape, all of which are influenced by the synthesis method and their experimental conditions [37, 50, 51]. Chemical, physical, or biological methods are common routes of synthesizing nanoscale materials, which are classified into two main categories: (i) top-down approach—nanomaterial obtained from bulk matter; and (ii) bottom-up approach—obtained from atomic scale, then integrated to nanomatter based on the principle of synthesis [10–12, 21, 24, 32, 35, 45, 52]. The schematic representation of the top-down approach and bottom-up approach is shown in Fig. 1 It is also necessary to designate a nanoparticle synthesis method, as conventional physical or chemical processes give rise to impediments and detrimental effects on ecology, such as elevated cost, time-consuming labor, complex equipment and synthesis parameters, complex high energy requirements, use of chemical compounds and templates, and generation of toxic waste, rendering them less biocompatible and undesirable for therapeutic applications [10, 15–17, 20, 21, 24, 25, 30–32, 35–39, 46, 49, 53–59].

These aroused concerns activated the consciences of an alternative synthesis producer with the ultimate goal of procuring a novel, less toxic, eco-sustainable technique: green synthesis, which furnishes environmentally acceptable products that are biocompatible, non-volatile, renewable, rapidly processed, cost-effective, low-energy input, more productive, eco-friendly solvent, low production cost, waste-minimizing, usage of non-toxic solvent, and time-efficient [1–3, 15, 20, 27, 28, 31, 32, 35, 36, 39, 41, 57, 59–66].

### Biosynthesis of nanoparticles

This biosynthesis approach has been explored more by researchers, as it produces minimal harm to the ecosystem by adopting the principles of green chemistry, which modify the chemical structure and enhance biocompatibility [31, 36, 39]. The biological synthesis technique [14], (green synthesis) belongs to the bottom-up strategy and involves the use of biological entities like plants and their parts, microbes, naturally derived substances, biopolymers, biomolecules, marine organisms, and natural by-products [2, 3, 10, 16]. These biological systems have the potential to synthesize nanoparticles through their biologically active secondary metabolites, which naturally reduce the ions to elemental metals by adhering, then subsequently growing through nucleation sites, followed by particle unification to form nanoparticles [19, 21, 29]. The nanoparticles are stabilized at a particular size reducing agglomeration, aggregation, or oxidation, and capping through bio-coating that strengthens their biocompatibility, making them suitable for medical application [32, 35, 36, 44, 46, 53, 54, 59, 63–69]. However, when treated with diverse biomolecules, each undergoes unique mechanics during the synthesis process, resulting in nanoparticles with distinct properties that depend on the bioextract’s chemical constitution’s nature and quantity that creates a change of interaction with precursors [27, 32, 36]. Biosynthesis is governed by various parameters such as pH, solvent type, temperature, and pressure [39]. The functional components present in biological extracts that reduce and stabilize nanoparticles include alcoholic groups, alkaloids, hydroxyls, polyphenols, terpenoids, flavonoids, carbonyls, amines, tannins, carbohydrates, proteins, vitamins, amino acids, and sugars [16, 27, 32, 33, 53, 70].

### Metal oxide nanoparticles as antimicrobials

**Fig. 2:**
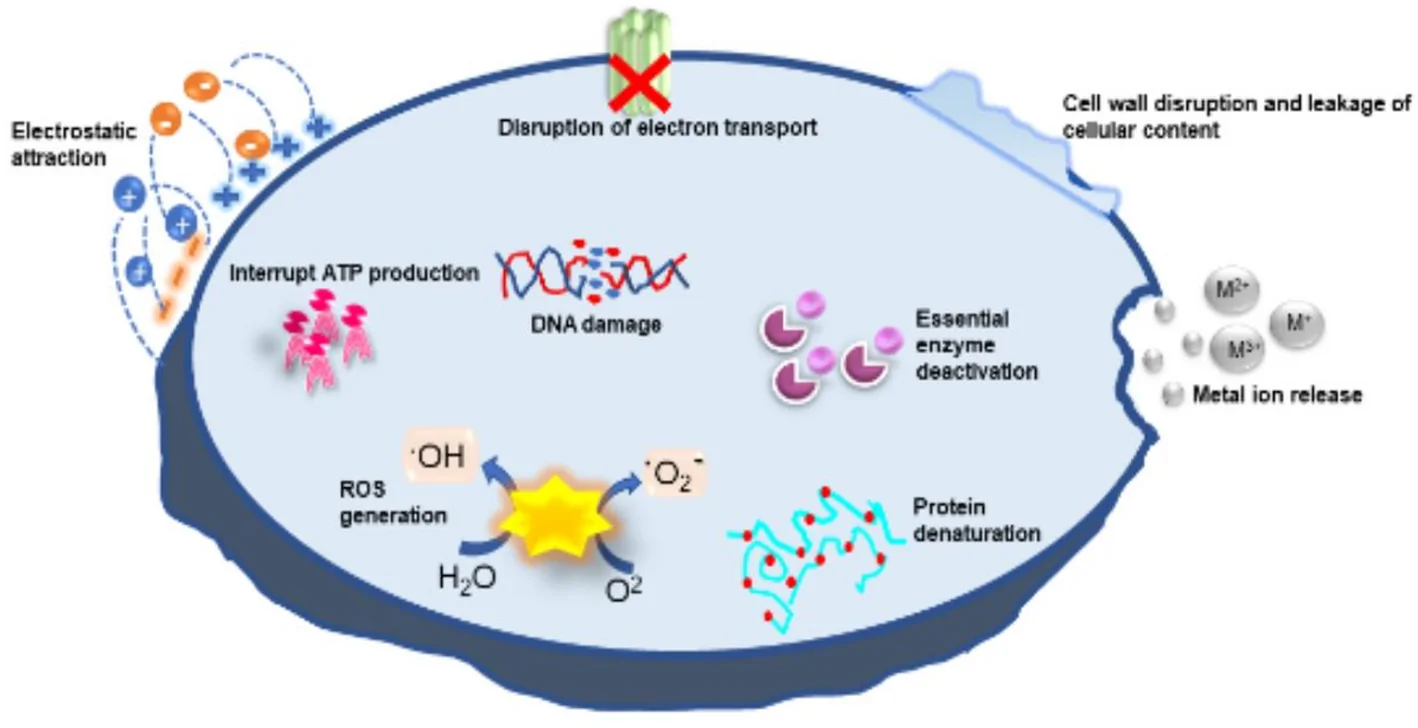
Schematic illustration of the possible mechanism of nanomaterials against pathogenic microorganisms

Metal oxides are produced when metallic elements combine with oxygen to form oxide compounds [71]. These metal oxides can exhibit metal, semiconductor, and insulating behaviour, and their significance increases; the combination of organic and inorganic nanoparticles with biomolecules enables the design of nanoparticles with desired functional properties for biomedical applications [72, 73]. The employment of metal oxide nanoparticles for the resistance aroused by microbes is due to their enhanced stability, tunable size and shape, surface charge, high surface ratio, cost-effectiveness, low toxicity, and a broad spectrum of antimicrobial activity [7, 57, 74]. Although the exact mechanism remains uncertain, lots of distinctive mechanisms have been reported for metal oxides as antimicrobial agents to exert a lethal effect on the pathogen, either by (i) metal ion release or (ii) internalisation of nanoparticles through electrostatic attraction, which causes a change in pH, then damages the pathogen’s cell membrane deoxyribonucleic acid (DNA) and inhibits the pathogen’s ability to synthesise its cell wall, solute transporters and nucleic acid, then deactivates energy metabolism, essential enzymes, adenosine triphosphate (ATP) production and dysfunction of protein or (iii) generation of reactive oxygen species (ROS), creating oxidation of proteins and essential enzymes, leakage of cellular components, and disruption of the electron transport chain, cell division, and the cell’s signals or (iv) producing genotoxic effects by interfering with genetic material replication, intracellular signalling, and genomic expression for bacterial microbes or (v) physical disruption of the cell membrane that leaks the cellular components [8, 10, 39, 63]. Figure 2 shows the antimicrobial mechanism taking place on interaction of nanomaterials against pathogenic microbes.

Exploration of biomolecule-assisted metal oxide nanoparticles is accelerating due to the quest for obtaining sustainable materials. Biosynthesised metal oxide is retained as it finds a role in the scientific implications of biomedical science because of special aspects like tunable physicochemical configuration, substantial surface area, nanoscale dimensions, crystallinity, low toxicity, high density, adhesiveness, controlled morphology, surface charge, cellular penetration, semiconducting properties, and intensified chemical and thermal stability [25, 39, 53, 54, 57, 62, 74–77]. Here, this review summarises the biosynthesis of metal oxides and multiple strategies to enhance activity against microbes, such as doping and nanocomposite formation. A comprehensive approach consolidates the usage of biomolecules of plant extract, microorganisms and other biologically derived materials, with the ultimate goal of promoting environmentally sustainable products. Furthermore, it discusses existing limitations on reproducibility, scalability, and standardisation of synthesis and green extract protocols and explores future prospects on advancing biosynthesised metal oxides for clinical translation.

### Literature search strategy

**Fig. 3:**
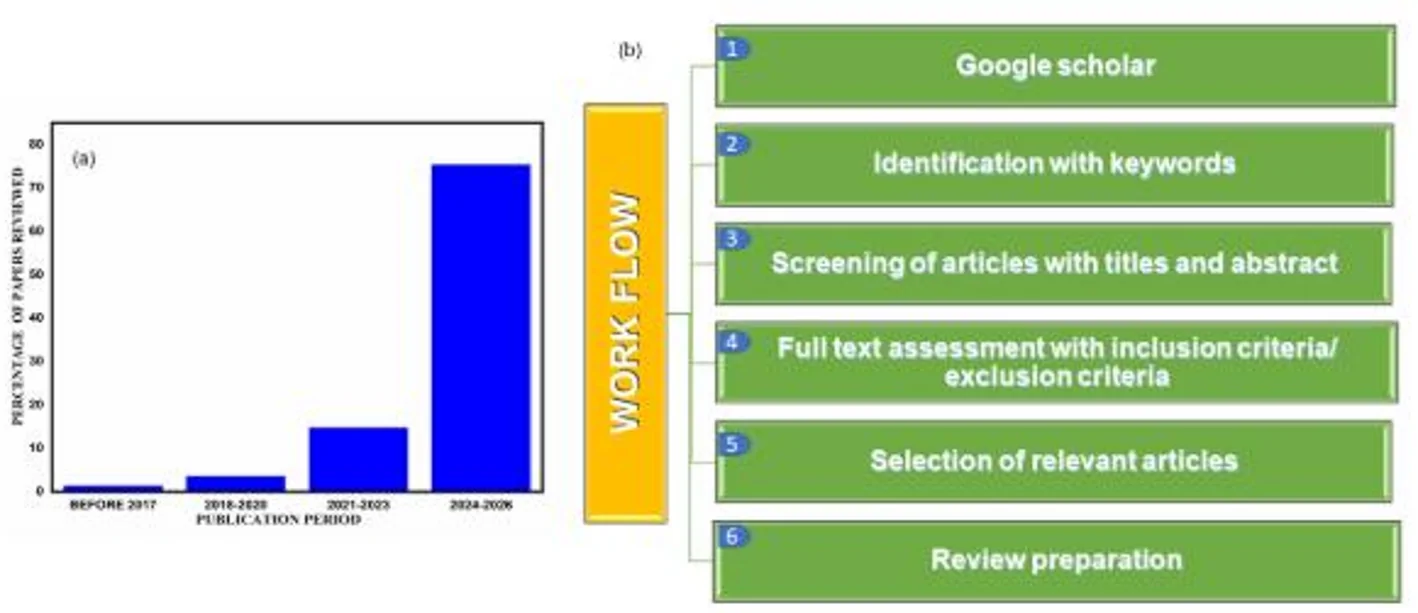
**a** The bar chart shows the publication year-wise distribution of the references included in this review. **b** This review article’s literature workflow schematic representation

In this review, the full-text articles collected were from the Google Scholar database and were mainly from journals published by Elsevier, Springer Nature, MDPI, Wiley, ACS Publications, RSC, Frontiers Media, and other recognized scientific publishers. The keywords used for the literature search were a combination of green synthesis, biosynthesis, metal oxide nanoparticles, and antimicrobials. The literature review revealed the current research findings and recent scientific developments in the area of green synthesis of metal and metal oxide nanoparticles for the period 2024–2026 Fig. 3a. However, it did not take into account the year restrictions for basic concepts and definitions concerning nanomaterials, metal oxide nanomaterials, green synthesis, the background of antibiotic resistance, and antimicrobial mechanisms. Data extraction for analysis was on precursor material, biological source, synthesis technique, physicochemical characterization and antimicrobial performance. The inclusion criteria were research and review articles of metal oxide nanoparticles, published in English, using plant-, microbial-, or biomolecule-assisted methods with physicochemical characterization analysis. Excluded were conference abstracts, theses, and patents unrelated to the antimicrobial application and green synthesis in bulk materials without nanoparticle characterization Fig. 3b.

## Biosynthesised metal oxide nanoparticles

### Plant-mediated biosynthesis of metal oxide nanoparticles

#### Role of plant extract phytochemicals in nanoparticle formation

Researchers have shifted towards green synthesis by using plants and their parts (flowers, stems, leaves, roots, fruits, seeds, latex, bark, calluses, peels, etc.), as this is simple and ecologically compatible [2, 30, 33, 36, 47, 52, 78–83] It offers benefits such as easy accessibility, safe handling, cost-effectiveness, eco-friendliness, reduced toxicity of byproducts, wide plant availability, less time for synthesis, and improved large-scale production, as its reservoir of abundant metabolite content has a significant role in nanoparticle formation along with metal precursors, including sugars, tannins, terpenoids, phenols, cardiac glycosides, alkaloids, flavonoids, and steroids. These phytochemical elements act as reducing agents and capping or stabilising agents through the functional groups present in them, such as allyl, alkoxy, amino, hydroxyl, carboxylic, and sulfhydryl [17, 39, 40, 52–55, 61]. The assistance of plant parts in the synthesis of metal oxide nanoparticles for medicinal and pharmaceutical applications was not doubtful, as plants have been known for their medicinal use since ancient times [28]. The plant-assisted nanoparticle’s quality and stability are shaped by metal salts, the concentration of phytochemicals, pH, and temperature. Additionally, the quantity of phenolic and polar components in the plant extract shows an effect on the properties and morphology of resultant nanomaterials [9, 30, 52, 61]. Table 1 Showed various metal oxides synthesised using plant extract and their impact on microbes.

**Table 1:** Representative studies on plant extract assisted metal oxide nanoparticles and its and antimicrobial efficacy

Metal oxide NPs	Plant Extract	Particle size & morphology	Microorganism	Antimicrobial activity	Reference
Aluminium oxide NPs	*Chrysophyllum albidum* *Terminalia catappa* *Mimusops Coriacea*	54.4 nm, 58.3 nm and 25.8 nm Quasi-spherical	*Staphylococcus aureus* *Bacillus subtilis* *Escherichia coli* *Pseudomonas aeruginosa* *Candida albicans* *Trichophyton rubrum*	**MIC** 0.125 mg/mL 0.250 mg/mL 12.5 mg/mL 25 mg/mL 3.125 mg/mL 6.25 mg/mL 2.1 mg/mL 1.5625 mg/mL 2.4 mg/mL 12.5 mg/mL 12.5 mg/mL 25 mg/mL 3.125 mg/mL 6.25 mg/mL 12.5 mg/mL 25 mg/mL 12.5 mg/mL 25 mg/mL	[15]
Cobalt oxide NPs	*Alhagi maroum*	78.92 nm Irregular	*Listeria monocytogenes* *Streptococcus pyogenes* *Staphylococcus aureus*	**ZOI-500 µg/mL** 31.66 ± 0.88 mm 24.33 ± 2.08 mm 25.66 ± 0.33 mm	[19]
CoO NPs	*Calotropis procera* Leaves	45 nm Spherical shape	*Staphylococcus aureus* *Pseudomonas aeruginosa*	**ZOI-10 µg/mL** 8 ± 1.2 mm 6 ± 0.8 mm **25 µg/mL** 14 ± 1.5 mm 12 ± 1.3 mm **50 µg/mL.** 20 ± 2.1 mm 22 ± 2.0 mm	[84]
Copper oxide NPs	Aloe vera	15.8 nm Sago-shaped morphology	*Listeria monocytogenes* *Klebsiella pneumoniae* *Salmonella enterica serovar Typhi* *Pseudomonas aeruginosa*	**ZOI-1 mg/mL** 15 ± 0.04 mm 10 ± 0.04 mm 13 ± 0.02 mm 10 ± 0.08 mm	[53]
CuO NPs	Resin extract of *Boswellia papyrifera*	35.8 ± 3.7 nm Near-spherical particles with spherical, cuboidal, and triangular forms	*Staphylococcus aureus* *Listeria monocytogenes* *Escherichia coli* *Klebsiella pneumoniae*	**ZOI-50 mg/mL** 18.40 ± 0.23 mm 6.91 ± 0.15 mm 8.92 ± 0.15 mm No activity **100 mg/mL** 22.86 ± 0.15 mm 11.03 ± 0.15 mm 11.11 ± 0.18 mm 4.91 ± 1.95 mm **200 mg/mL** 27.21 ± 0.12 mm 17.51 ± 0.24 mm 13.81 ± 0.18 mm 6.80 ± 0.25 mm	[85]
CuO NPs	*Nerium oleander*	23.7 nm Mild agglomerated quasi spherical	*Staphylococcus aureus* *Escherichia coli* *Bacillus subtili* *Salmonella typh*	**(ZOI- -)** 18.6 mm 17.1 mm 16.4 mm 15.2 mm	[86]
CuO NPs	*Ficus carica* leaves	13.92 nm to 56.10 nm Spherical to elliptical	*Escherichia coli* *Proteus mirabilis* *Streptococcus mutans*	**ZOI-100 µg/ml** 16.87 ± 3.42 mm 17.78 ± 2.09 mm 20.32 ± 4.31 mm **ZOI-50 µg/ml** 15.56 ± 3.87 mm 16.21 ± 3.19 mm 19.19 ± 2.78 mm **ZOI-25 µg/ml** 14.25 ± 2.81 mm 15.09 ± 3.05 mm 18.03 ± 4.08 mm **ZOI-12.5 µg/ml** 13.19 ± 2.65 mm 14.29 ± 2.81 mm 17.32 ± 5.25 mm	[87]
CuO NPs	*Blighia sapida* seed pod extract	6.21 nm Oval and plateletlike surface morphologies	*Escherichia coli* *Pseudomonas aeruginosa* *Klebsiella pneumoniae* *Proteus penneri* *Staphylococcus aureus* *Streptococcus pyogenes*	**With 2**,** 4**,** 6**,** and 8 mg/l** Higher antimicrobial activity against Gram-negative microbe	[88]
CuONPs	*Coffea arabica* leaves	35 and 45 nm Cuboid shape	*Escherichia coli*	**ZOI-30 µg/disc** 10 mm	[89]
CuO NPs	*Amomum subulatum* Fruit Extract	26.04 nm Spherical, polydisperse with aggregates	*Staphylococcus aureus* *Escherichia coli* *Bacillus subtilis*	**ZOI-250 µg/mL** **100 µL** 14 ± 0.15 mm 14.5 ± 0.05 mm 14 ± 0.12 mm **50 µL** 10 ± 0.2 mm 10 ± 0.12 mm 10 ± 0.11 mm	[90]
CuO NPs	*Mentha* leaf extract	19.08 nm -	*Escherichia coli* *Staphylococcus aureus* *Candida albicans*	**(ZOI- -)** 1.4 nm 1.3 nm 1.5 nm	[91]
CuO NPs	*Aerva* *javanica* Leaf Extract	50.1 nm Spherical morphology	*Escherichia coli* *Pseudomonas aeruginosa* *Staphylococcus aureus* *Acinetobacter baumannii*	**ZOI-50 µg/mL** 5 ± 1 mm 5 ± 1 mm 9 ± 1 mm 5 ± 1 mm	[92]
CuO NPs	Fruit extract of *Piper* *cubeba*	56.8 nm Spherical with some aggregations	*Escherichia coli* *Proteus vulgaris* *Bacillus subtilis* *Staphylococcus aureus* *Salmonella typhimurium*	**ZOI-100 µg/mL** 19 + 0.3 mm 21 + 0.2 mm 20 + 0.5 mm 21 + 0.6 mm 21 + 0.3 mm	[93]
CuO NPs	Orange peel	17 nm Irregular crystals	*Escherichia coli* *Staphylococcus aureus* *Klebsiella penumoniae* *Bacillus subtilis* *Fusarium oxysporum* *Aspergillus * *avus* *Aspergillus nigar*	**MIC** 2500 µg/mL 1500 µg/mL 2000 µg/mL 1500 µg/mL 2500 µg/mL 3000 µg/mL 3000 µg/mL	[94]
CuO NPs	Orange peel extract	13.84 nm Granular morphology	*Bacillus cereus* *Staphylococcus aureus* *Escherichia coli* *Pseudomonas aeruginosa* *Candida albican*	**MIC** 0.625 µg/µL 0.313 µg/µL 1.250 µg/µL 5.000 g/µL 5.000 µg/µL	[95]
CuO NPs	Tragacanth gum	21 nm -	*Salmonella typhimurium* *Escherichia coli* *Pseudomonas aeruginosa* *Proteus mirabilis* *Klebsiella pneumonia* *Enterococcus faecalis* *Staphylococcus aureus*	**MIC** 1 µg/mL 1 µg/mL 1.5 µg/mL 1 µg/mL 1 µg/mL 1 µg/mL 1 µg/mL	[96]
CuO NPs No calcination (CT0) calcined 200 ◦C (CT1), 300 ◦C (CT2) 500 ◦C (CT3)	*Camellia sinensis* extract	5.9 nm 9.2 nm 11.5 nm 15.5 nm Irregular spherical morphology	*Staphylococcus aureus* *Streptococcus mutans* *Klebsiella pneumoniae* *Escherichia coli*	**ZOI-1.87 mg** 11 mm 10 mm 11 mm 9 mm 15 mm 17 mm 20 mm 10 mm 11 mm 10 mm 12 mm 10 mm 24 mm 22 mm 29 mm 22 mm	[97]
CuO NPs (50% of Cu) nanoparticles	*Rosmarinus officinalis*	18–26 nm Spherical	*Escherichia coli* *Staphylococcus aureus* *Candida albicans* *Bacillus cereus*	**ZOI-200mg** 26.0 ± 0.10 mm 25.0 ± 0.15 mm 18.1 ± 0.32 mm 17.0 ± 0.0 mm	[55]
CuO/MgO nanocomposite	*Citrus limonium* extract	44 nm Uneven particles with dense agglomerated morphology	*Staphylococcus epidermidis* *Escherichia coli*	**ZOI-150 µg/mL** 27.4 ± 0.15 mm 25.2 ± 0.1 mm **100 µg/mL** 18.2 ± 0.20 mm 19.2 ± 0.1 mm **50 µg/mL** 16.5 ± 0.36 mm 15.3 ± 0.1 mm	[98]
FeO NPs	*Hibiscus sabdariffa*	61 nm Spherical and coarse needle	*Klebsiella pneumoniae* *Escherichia coli* *Pseudomonas aeruginosa*	**(ZOI- -)** 18 mm 16 mm 14 mm	[3]
FeO NPs	*Cymbopogon citratus*	5–10 nm Slightly spherical with porous morphology	*Escherichia coli*	**ZOI-100 µl** 17 ± 0.56 mm	[80]
Fe_2_O_3_ NPs	*Thymus* * migricus*	14.47 nm Spherical nanoparticles	*Staphylococcus aureus* *Salmonella enterica*	**ZOI-400 µg mL-1** 16.15 ± 0.19 mm 13.31 ± 0.46 mm	[99]
Fe_2_O_3_ NPs	*Cissus rotundifolia*	10.06 nm Spherical and irregular	*Pseudomonas aeruginosa*	**ZOI-100 µg/mL** 11 mm	[100]
Fe_3_O_4_ NPs	*Qazwan* Seeds Extract	16–50 nm Spherical	*Escherichia coli*	**ZOI-1.0 mg/mL** 10 mm **7.0 mg/mL** 14 mm **10.0 mg/mL** 16 mm **15.0 mg/mL** 18 mm **20.0 mg/mL** 18 mm	[101]
Iron oxide NPs	*Salvia* *officinalis* leaf extract	42.327 nm Spherical	*Escherichia coli* *Pseudomonas aeruginosa* *Staphylococcus aureus*	**ZOI-100 µg/ disk** 20.56 ± 0.62 mm 21.14 ± 0.16 mm 17.26 ± 0.26 mm	[102]
Iron oxide NPs	Cape gooseberry plant leaf	26.61 nm Near-spherical particles	*Streptococcus agalactiae* *Staphylococcus aureus* *Salmonella enterica* *Pseudomonas aeruginosa*	**ZOI-10 mg/ml** 17 mm 24 mm 19 mm 18 mm	[52]
Iron oxide NPs	*Hedychium spicatum* Leaf *Hedychium spicatum* leaf rhizome	Nearly spherical-shaped nanoparticles	*Escherichia coli* *Staphylococcus aureus*	**ZOI-500 µg/mL** 33 nm 32 mm 31 mm 29 mm	[103]
Iron oxide NPs	Bark extract of *Soymida febrifuga*	90 nm Polyhedral morphology	*Clavibacter michiganensis* *Enterococcus faecium* *Escherichia coli* *Pseudomonas aeruginosa*	**ZOI-25 µg/mL** 12.7 ± 0.3 mm 14 ± 0.2 mm 16.8 ± 0.5 mm 17.6 ± 0.5 mm **50 µg/mL** 4.3 ± 0.4 mm 17.3 ± 0.6 mm 18 ± 0.4 mm 19.4 ± 0.5 mm **75 µg/mL** 15.7 ± 0.2 mm 19.8 ± 0.3 mm 21.1 ± 0.9 mm 22.4 ± 1.2 mm **100 µg/mL** 17.3 ± 0.4 mm 21.4 ± 0.6 mm 23.7 ± 0.2 mm 24.6 ± 0.4 mm	[104]
Iron oxide NPs	*Myristica fragrans* leaf extract	25 to 45 nm Quasi-spherical shape	*Escherichia coli* *Bacillus subtilis*	**ZOI-1 mg/mL** 12 mm 9 mm	[105]
Iron oxide NPs	*Bombax malabaricum*	09 ± 2 nm -	*Staphylococcus aureus* *Bacillus halodurans* *Micrococcus leutus* *Escherichia coli* *Aspergillus niger* *Aspergillus flavus*	**ZOI-20 µL** 17.66 ± 0.33 mm 13.30 ± 0.178 mm 28.10 ± 0.106 mm 23.18 ± 0.267 mm 19.17 ± 0.121 mm 15.16 ± 0.14 mm	[106]
Iron oxide NPs	*Nigella sativa* seed extract	31.45 nm Spherical	*Escherichia coli* *Staphylococcus aureus*	**ZOI- 0.1 mg·mL − 1** 12.34 ± 0.58 mm 11.52 ± 0.58 mm	[107]
Iron oxide NPs	*Hibiscus rosa* *sinensis Flowers*	36.4 nm Polydispersity shapes	*Staphylococcus aureus* *Pseudomonas aeruginosa* *Klebsiella pneumonia* *Escherichia coli*	**(ZOI- -)** Range 2 to 6 mm	[108]
Iron oxide NPs	*Psidium guajava L.* *Leaves*	21.75 nm Large agglomerated clusters	*Shigella sonnei* *Staphylococcus aureus* *Candida tropicalis* *Candida albicans*	**(ZOI- -)** 13 mm 13 mm 15 mm 13 mm	[109]
Iron oxide NPs	*Quercus Infectoria Galls*	3.7 nm	*Staphylococcus aureus* *Escherichia coli*	**MIC** > 1000 > 1000	[110]
Iron oxide NPs	Green coffee	Pyramidal morphology	*Staphylococcus aureus* *Escherichia coli*	**MIC** 62.5 µg/ml 125 µg/ml	[111]
Iron oxide NPs (Iron and extract- 01:01, 02:04,01:04).	Black cumin seed	4–16 nm	*Streptococcus mutans* *Escherichia coli* *Aspergillus fumigatus* *Aspergillus niger*	**ZOI-150 µL** **01:04–12 mm** 13 mm 16 mm 14 mm **02:04–14 mm** 15 mm 10 mm 16 mm	[112]
Hydroxyapatite-Fe_3_O_4_ NCs (HA)_1−x_ (Fe_3_O_4_) _x_	*Ocimum sanctum*	17–31 nm Agglomerated spherical	*Escherichia coli* *Staphylococcus aureus*	**ZOI-250 µg/mL** 12 mm 13.83 mm	[113]
MgO NPs	Orange peel extract	9.08 nm irregular form	*Bacillus cereus* *Staphylococcus aureus*	**MIC** 0.600 µg/µL 0.650 µg/µL	[114]
MnO_2_ NPs	*Sapindus mukorossi*	16 nm Irregular spherical	*Staphylococcus aureus* *Bacillus subtilis* *Pseudomonas aeruginosa* *Escherichia coli*	**ZOI-50 µg** 5.3 mm **50 µg** 7.3 mm **50 µg** 8.3 mm **250 µg** 9 mm	[58]
Ag-MgO NPs	*Morinda tinctoria*	21 nm Rod shaped	*Staphylococcus aureus* *Escherichia coli*	**ZOI-5 µg/mL** 6 mm 5 mm **10 µg/mL** 8 mm 7 mm **15 µg/mL** 12 mm 10 mm **20 µg/mL** 15 mm 14 mm	[56]
Ag@Sm_2_O_3_/rGO NCs	*Punica Granatum*	10–20 nm Agglomrerated Sm_2_O_3_ attached to rGO sheet	*Escherichia coli*	Sm_2_O_3_ ZOI-11.23 ± 0.42 mm NCs – No activity	[115]
Ag-doped SnO_2_ NPs Ag_1−x_ NO_3_ (x = 0.2, 0.4, 0.6, 0.8, and 1.0) nanoparticle	*Zingiber officinale*	9.59–16.79 nm Agglomerated irregular	*Staphylococcus aureus* *Escherichia coli* *Fusarium oxysporum* *Fusarium graminearum*	**(ZOI- -)** 0.8Ag-doped SnO2 > 0.6Ag-doped > 0.4Ag-doped > 0.2Agdoped > undoped SnO_2_ NPs	[2]
SnO@FeCl_2_- NPs	*Olea europaea*	28.5 nm	*Staphylococcus aureus* *Pseudomonas aeruginosa* *Proteus vulgaris* *Escherichia coli* *Klebsiella pneumonia*	**ZOI-5 mg/ml** 15.5 ± 0.6 mm 17.6 ± 0.3 mm 19.2 ± 0.7 mm 21.2 ± 0.9 mm 12.3 ± 0.6 mm	[29]
TiO_2_ NPs	*Syringodium isoetifolium*	2 to 50 nm Spherical morphology	*Staphylococcus epidermidis* *Pseudomonas aeruginosa*	**ZOI-50 µg** 18.6 mm 17.6 mm	[116]
TiO_2_ NPs	*Cocos nucifera* pollen extract	17.4 nm Spherical shape	*Escherichia coli* *Staphylococcus aureus*	**ZOI-50 µL** 18.5 ± 0.32 mm 16 ± 0.12 mm **100 µL** 21.0 ± 0.23 mm 17.5 ± 0.45 mm **150 µL** 24.5 ± 0.30 mm 19.8 ± 0.21 mm **200 µL** 27.3 ± 0.31 mm 21.5 ± 0.10 mm **250 µL** 29.5 ± 0.12 mm 23.0 ± 0.31 mm	[117]
Ag-doped TiO_2_ NPs	*Caesalpinia pulcherrima*	24 nm Spherical shape	*Staphylococcus aureus* *Escherichia coli* *Candida albicans* *Pseudomonas aeruginosa*	11 ± 0.33 mm 10 ± 0.11 mm 14 ± 0.66 mm 14 ± 0.33 mm	[60]
Zn doped TiO_2_ NPs	*Zanthoxylum armatum*	15 nm Spherical	*Salmonella* *Escherichia coli* *Staphylococcus epidermidis* *Pseudomonas aeruginosa* *Aspergillus flavus* *Aspergillus niger* *Candida alabrata* *Candida ablicans*	**ZOI-30 mg/ml** 23 mm 21 mm 19 mm 22 mm 22 mm 19 mm 23 mm 21 mm	[38]
Ag@WO_3_/rGO NCs	*Punica Granatum Acalypha*	37 nm Wrinkled sheet	*Escherichia coli*	**ZOI**-undoped WO_3_ 10.12 ± 0.42 mm Ag doped – No activity	[118]
ZnO NPs	*Alendula officinalis*	28.23 nm Agglomerated spherical	*Staphylococcus aureus* *Klebsiella pneumoniae* *Escherichia coli*	**ZOI-50 µg/ml** 22.8 ± 0.3 mm 12.4 ± 0.3 mm 10.6 ± 0.6 mm **75 µg/ml** 23.6 ± 0.7 mm 16.8 ± 0.6 mm 12.7 ± 0.8 mm **100 µg/ml** 35.2 ± 0.9 mm 23.6 ± 0.1 mm 13.5 ± 0.1 mm	[24]
ZnO NPs	*Chenopodium exsuccum*	21 nm Elongated pseudospherical	*Escherichia coli* *Klebsiella pneumonia* *Staphylococcus aureus* *Micrococcus luteus*	**ZOI-0.5 mg/mL** 4.1 mm 7.1 mm 11.7 mm 12.9 mm **1 mg/mL** 5.7 mm 9.80 mm 15.7 mm 16.9 mm **2 mg/mL** 3.8 mm 6.5 mm 10.3 mm 11.2 mm **4 mg/mL** 4.9 mm 7.3 mm 12.8 mm 9.1 mm	[46]
ZnO NPs	Acorn Fruit	29.6 nm Asymmetrical clusters	*Escherichia coli* *Staphylococcus aureus*	**ZOI-10 µg/ml** 15 ± 02 mm 17 ± 03 mm **20 µg/ml** 18 ± 04 mm 20 ± 06 mm **40 µg/ml** 21 ± 03 mm 22 ± 03 mm **80 µg/ml** 24 ± 05 mm 26 ± 02 mm	[119]
ZnO NPs	*Thevetia peruviana* flowers	27.84 nm Nanocrystalline spherical particles	*Staphylococcus aureus* *Escherichia coli*	**ZOI-20 µg/mL** 18.2 ± 1 mm 20.7 ± 2 mm **50 µg/mL** 20.4 ± 1 mm 22.1 ± 2 mm **100 µg/mL** 40.1 ± 2 mm 45.2 ± 1 mm	[120]
ZnO NPs	Date palm seed powder	63.73 nm Hexagonal morphology	*Bacillus cereus* *Escherichia coli* *Staphylococcus aureus*	**ZOI-0.5 mg/mL** 1.33 ± 0.57 mm 4.33 ± 1.15 mm 8.66 ± 1.15 mm **1 mg/mL** 8.66 ± 0.57 mm 13.00 ± 1.00 mm 11.00 ± 1.00 mm **2 mg/mL** 10.00 ± 1.00 mm 14.00 ± 0.00 mm 13.66 ± 1.53 mm **4 mg/mL** 11.67 ± 1.53 mm 15.00 ± 1.00 mm 17.00 ± 1.00 mm	[121]
ZnO NPs	Leaf extract of *Euphorbia lateriflora*	4–5 nm Spherical particles with smooth surfaces	*Klebsiella pneumoniae* *Escherichia coli* *Pseudomonas aeruginosa*	**(ZOI- -)** 27.00 ± 2.42 mm 21.00 ± 1.83 mm 17.00 ± 1.25 mm	[122]
ZnO NPs	*Phoebe zhennan *and *Croton tiglium* leaves	11.3 nm Spherical morphology	*Enterococcus faecalis* *Enterobacter cloacae*	**ZOI-25 µg/mL** 21.6 mm 21 mm **50 µg/mL** 26.3 mm 25 mm **75 µg/mL** 28 mm 27 mm **100 µg/mL** 31.6 mm 32.66 mm	[123]
ZnO NPs	*Moringa oleifera* leaf extract	20.7 nm Quasi-spherical to irregularly shaped nanoparticles	*Staphylococcus aureus* *Escherichia coli*	**(ZOI- -)** 20.0 mm 21. 2 mm	[124]
ZnO NPs	*Zea mays leaf extract* Extract concentration 1%	10.86 nm Spherical and semispherical	*Escherichia coli* *Pseudomonas aeruginosa* *Klebsiella pneumoniae*	**ZOI-50** ***µ*** **L** 14 ± 1.2 mm 12.66 ± 0.57 mm 12.66 ± 0.57 mm	[125]
ZnO NPs	*Typha domingensis* leaf extract	26.74 nm Agglomerated spherical	*Colletotrichum sp.* *Fusarium sp.*	**ZOI-1.0 mg/mL** 13 ± 0.52 mm 14 ± 0.56 mm **1.5 mg/mL** 14 ± 0.56 mm 15 ± 0.60 mm **2.0 mg/mL** 20 ± 0.80 mm 17 ± 0.68 mm	[126]
ZnO NPs	Aloe vera	7.34–16.32 nm Predominantly spherical	*Escherichia coli* *Bacillus subtilis* *Candida albicans*	**ZOI-100 mg/mL** 1.8 mm 2.0 mm 1.9 mm	[127]
ZnO NPs	*Platycladus orientalis*	26.9 nm irregularly shaped particle high agglomeration	*Listeria monocytogenes* *Escherichia coli* *Enterococcus faecalis*	**ZOI-6.4 mg/mL** 10.65 ± 0.02 mm 11.25 ± 0.13 mm 10.45 ± 0.12 mm **3.2 mg/mL** 9.55 ± 0.13 mm 9.50 ± 1.05 mm 9.25 ± 0.08 mm **1.6 mg/mL** 8.67 ± 0.02 mm 8.37 ± 0.05 mm 8.25 ± 0.04 mm **0.8 mg/mL** 5.40 ± 0.24 mm 6.15 ± 0.27 mm 5.05 ± 0.17 mm	[128]
ZnO NPs	Triphala	26.26 nm to 111.67 nm Interconnected network of nanoparticles	*Staphylococcus aureus* *Escherichia coli* *Pseudomonas aeruginosa* *Candida albicans*	**ZOI-1 mg/mL** 18 mm 12.33 mm 15.67 mm 12.33 mm	[129]
ZnO NPs	*Duranta erecta* leaves	25 nm Agglomerated structures	*Escherichia coli* *Pseudomonas aeruginosa* *Bacillus subtilis*	**ZOI-250 µg/mL** 18.11 ± 0.61 mm 21.46 ± 0.14 mm 17.40 ± 0.89 mm **500 µg/mL** 22.19 ± 0.76 mm 23.33 ± 0.24 mm 19.50 ± 0.25 mm **750 µg/mL** 27.01 ± 0.21 mm 25.89 ± 0.32 mm 20.10 ± 0.14 mm **1000 µg/mL** 29.18 ± 0.16 mm 28.33 ± 0.84 mm 20.98 ± 0.32 mm	[130]
ZnO NPs	Lychee peel	2.18 nm Roughly spherical and crowded	*Pseudomonas aeruginosa* *Bacillus subtilis* *Staphylococcus aureus* *Escherichia coli*	**(ZOI–)** 23.0 mm 22.0 mm 17.0 mm 15.0 mm	[131]
ZnO NPs	*Punica granatum* Peels	43 nm Spherical	*Bacillus subtilis* *Staphylococcus aureus* *Pseudomonas aeruginosa* *Escherichia coli* *Candida albicans*	**ZOI-200 µg mL** ^**− 1**^ 17.7 ± 0.6 mm 17.3 ± 0.6 mm 24.7 ± 0.7 mm 19.3 ± 0.6 mm 17.0 ± 0.1 mm	[132]
ZnO NPs	*Plantain* *peel *extracts	18.06 nm Spherical	*Salmonella enterica*, *Klebsiella pneumoniae*, *Staphylococcus aureus* *Bacillus cereus*	**(ZOI- -)** 10.67 ± 0.58 mm 12.00 ± 1.00 mm 27.67 ± 6.81 mm 12.00 ± 1.73 mm	[133]
ZnO NPs	*Brassica oleracea var.* *botrytis* leaf extract	52 nm Flower morphology	*Bacillus subtilis* *Staphylococcus aureus* *Klebsiella pneumoniae* *Escherichia coli*	**ZOI-100 µg/mL** 16 mm 13 mm 13 mm 9 mm	[134]
ZnO NPs	*Dillenia indica* leaf extract	25.47 nm Spherical morphology	*Staphylococcus aureus* *Listeria monocytogenes* *Escherichia coli* *Salmonella enterica* *serovar Abony* *Candida albicans*	**MIC** 0.625 mg/mL 0.313 mg/mL 10 mg/mL 5 mg/mL 10 mg/mL	[135]
ZnO NPs	Orange peel extract	22.81 nm Granular morphology	*Bacillus cereus* *Staphylococcus aureus* *Escherichia coli* *Pseudomonas aeruginosa* *Candida albicans*	**MIC** 0.625 µg/µL 0.625 µg/µL 0.625 µg/µL 10.000 µg/µL 5.000 µg/µL	[95]
ZnO NPs	*Artemisia absinthium L.*	36.6 nm spherical morphology	*Staphylococcus aureus* *Escherichia coli* *Candida albicans*	**MIC** 0.312 ± 0.00 mg/mL 1.25 ± 0.00 mg/mL 0.625 ± 0.00 mg/mL	[136]
ZnO-CuO nanocomposite	*Aegle marmelos* aqueous leaf extract	32.78 nm Densely packed particles with no distinct shape	*Staphylococcus aureus* *Escherichia coli*	**ZOI-0.5 µg/mL** 23.0 ± 0.00 mm 18.5 ± 0.70 mm	[137]
Bb-ZnO NPs	*Barleria buxifolia*	2–50 nm Spherical	*Escherichia coli* *Pseudomonas aeruginosa* *Staphylococcus aureus*	**ZOI-100 µg/mL** 23.31 ± 1.42 mm 20.17 ± 1.08 mm 16.46 ± 1.03 mm	[44]
Co-ZnO NPs	*Platycladus orientalis*	19.0 nm Irregular shaped with less aggregated morph	*Listeria monocytogenes* *Escherichia coli* *Enterococcus faecalis*	**ZOI-6.4 mg/mL** 13.50 ± 0.16 mm 13.65 ± 0.24 mm 14.05 ± 0.21 mm **3.2 mg/mL** 12.00 ± 0.27 mm 13.00 ± 0.24 mm 13.05 ± 0.19 mm **1.6 mg/mL** 10.50 ± 0.24 mm 10.60 ± 0.37 mm 11.20 ± 0.33 mm **0.8 mg/mL** 8.55 ± 0.09 mm 8.65 ± 0.21 mm 9.05 ± 0.25 mm	[128]
Cu–ZnO@ZrO_2_ nanoneedles	*Momordica charantia*	13 nm width and 170 nm length Needle like structure	*Staphylococcus aureus* *Escherichia coli*	**ZOI-50 µL** 3 mm 5.1 mm **100 µ**L 4.3 mm 4 mm **150 µL** 5.5 mm 4 mm **200 µL** 6.1 mm 6 mm	[138]
Sen–ZrO₂ NPs	*Cassia angustifolia* leaf extract	31.6–39.5 nm spherical morphology	*Staphylococcus aureus* *Enterococcus faecalis* *Escherichia coli* *Klebsiella pneumoniae*	**ZOI-100 µg** 16.0 ± 1.0 mm 15.0 ± 1.0 mm 11.0 ± 0.9 mm 10.0 ± 0.8 mm	[139]
Yttrium doped Zn NPs	*Murraya koenigii*	20 nm Spherical	*Bacillus subtilis* *Escherichia coli*	**ZOI-5 mg.mL-1** 24 mm 26 mm **10 mg.mL-1** 29 mm 25 mm **20 mg.mL-1** 30 mm 34 mm	[140]

#### Plant extract-derived metal oxide nanoparticles

Moawad et al., produced cobalt oxide NP particles sized at 78.92 nm in the assistance extract of *Alhagi maroum* with irregular morphology on surface analysis, with peaks at 840 cm⁻¹, 790 cm⁻¹, 3095 cm⁻¹, 1485 cm⁻¹, 560 cm⁻¹, and 660 cm⁻¹ due to C = H bend, 1,4 subs C–C stretch, N = C=O stretch, C-H stretch, and aryl, and also confirmed the cobalt oxide NP formation with peaks at 260 nm and 540 nm on Ultraviolet–Visible Spectroscopy (UV) analysis under the range of 200 to 800 nm. Then it was subjected to antibacterial activity against pathogenic bacteria: *Listeria*,* Streptococcus*,* Staphylococcus aureus*, and *Escherichia coli*, by the agar well diffusion method under ranging concentrations of 10, 20, 30, 40, 70, and 100 µg/mL of cobalt oxide NPs, which did not show effectiveness, and on increasing the concentration to 500 µg/mL, the zone of inhibition was 31.66 ± 0.88 mm, 24.33 ± 2.08 mm, 25.66 ± 0.33 mm, and 33.00 ± 6.08 mm and didn’t produce action against fungal strain. The cytotoxicity test against SKVO3 cells that belong to ovarian cancer by the Sulforhodamine B Assay (SRB) at concentrations of 0.7, 7, and 70 µg/mL gave 97.9561 ± 0.85, 80.4586 ± 1.78, and 27.667 ± 2.32% cell viability with a Half Maximal Inhibitory Concentration (IC_50_) value of 24.024 µg/mL [19].

Moroda et al., investigated through phytochemical tests and found that leaf extracts of *Rosmarinus officinalis* identified the presence of tannins, flavonoids, terpenoids, alkaloids, cardiac glycosides, and phenols that act as reducing agents and capping agents. They were subjected to the synthesis of copper oxide NPs with varying weight percentages of copper nitrate (10 w/w%, 20 w/w%, 30 w/w%, 40 w/w%, 50 w/w%, and 60 w/w%). The red shift was observed along with an increase in band gap on increasing concentration of CuO due to quantum confinement and a decrease in crystallite size from 26 to 19 nm with a monoclinic structure of spherical shape with little agglomeration, and the mechanism of antimicrobials was the interaction with ions of Cu and cell penetration, forming reactive oxygen species that destroy DNA and proteins that lead to death, or Cu ions damaging the cell wall in correspondence to the surface charge that caused protein denaturation and cell death, or Cu ions, after entering the cell, creating damage to DNA’s structure. The antimicrobial activity of CuO NPs by the disc diffusion method showed the zone of inhibition increased with an increase in concentration, except at 50% of CuO, which was 26.0 ± 0.10 mm, 25.0 ± 0.15 mm, 17.0 ± 0.0 mm, and 18.1 ± 0.32 mm for *Escherichia coli*,* Staphylococcus aureus*,* Bacillus cereus*, and *Candida albicans*, and proved its effectiveness even at low doses and had Minimum Inhibitory Concentration (MIC) values of 3.12 mg/mL with 2.2 ± 0.20 mm and 1.9 ± 0.25 mm zones of inhibition for *Staphylococcus aureus* and *Candida albicans* and 1.56 mg/mL with 0.2 ± 0.20 mm for *Escherichia coli* [55].*Macroptilium lathyroides* (L) extract was tested on phytochemical tests like Salkowski’s, Fehling’s, ferric chloride, sodium hydroxide, Molisch’s, lead acetate, Mayer’s, Valser’s, frothing, xanthoproteic, alcohol, and turbidity tests to test the presence of terpenoids, carbohydrates, tannins, flavonoids, phenols, alkaloids, saponins, proteins, amino acids, mucilage, and resins, according to Prabu et al., These presences were detected and further utilized in the synthesis of copper oxide NPs that were effective agents against many biomedical applications. They were polycrystalline-natured copper oxide NPs from *Macroptilium lathyroides (L*) extract of 7.3 nm in size with granulated spherical shapes and atomic percentages of Cu 31.18% and O 68.82% and weight percentages of Cu 64.28% and O 35.72%. Through Energy Dispersive X-ray Spectroscopy (EDAX) and X-ray Photoelectron Spectroscopy (XPS) analysis peaks occurred for Cu₂p, O₁s, and C₁s. Then the tested antimicrobial efficiency by the disc diffusion method with Amphotericin B revealed good efficiency against *Staphylococcus aureus* bacteria with 14 mm, 13 mm, and 9 mm and for *Escherichia coli* as 12 mm, 11 mm, and 11 mm at three concentrations (1000 µg/mL, 750 µg/mL, and 500 µg/mL) by Cu ions entering into the pathogen’s cell wall and denaturing by binding to oxygen, protein, sulfur, DNA, phosphorus, and nitrogen and for the *Candida albicans* fungus, 15 mm, 15 mm, and 7 mm with 1000 µg/mL, 750 µg/mL, and 500 µg/mL. The VERO cell line, by 3-(4,5-Dimethylthiazol-2-yl)-2,5-diphenyl tetrazolium bromide assay (MTT), got an IC_50_ value of 71.08 µg/mL, and viability percentages were 96.6%, 91.1%, 85.5%, 80.0%, 74.4%, 69.1%, 63.5%, and 58.0% at concentrations of 7.8, 15.6, 31.2, 62.5, 125, 250, 500, and 1000 µg/mL, and against the MCF 7 cell line, IC_50_ was 71.22 µg/mL at concentrations of 7.8, 15.6, 31.2, 62.5, 125, 250, 500, and 1000 µg/mL, and its viability was 74.03%, 66.84%, 59.64%, 52.28%, 45.08%, 37.89%, 30.89%, and 23.50%; and the antioxidant activity by 2,2-Diphenyl-1-picrylhydrazyl (DPPH) assay IC_50_ value was 86.96 µg/mL [67].

Flieger et al., used plant hops (*Humulus lupulus L.*,* Cannabaceae*) due to the presence of α acids-humulones and β-acids-lupulones that contributed to antimicrobial activity in the synthesis of iron oxide NPs with different solvents towards antimicrobial activity with different extracts (Dimethyl sulfoxide (DMSO), ethanol, acetone, and water) against Gram-positive bacteria to have the highest activity for the DMSO extract. The synthesized nanoparticles showed aggregation and agglomeration that was found due to van der Waals and magnetic forces, whereas Scanning Electron Microscopy (SEM) showed DMSO solvent decreased aggregation. The synthesized nanoparticles on Fourier Transform Infrared Spectroscopy (FTIR) analysis showed peaks at 3425 and 1631 cm⁻¹ denoting -OH groups; 1342, 938, and 830 cm⁻¹ are nitrate groups; and 620 and 547 cm⁻¹ correspond to the Fe-O bond, whereas the aqueous and alcoholic extracts band also showed the presence of polysaccharides, proteins, and phenolic compounds along with extra peaks at 3305 cm⁻¹ and 3195 cm⁻¹, 2960–2853 cm⁻¹, and 1440–1350 cm⁻¹ corresponding to stretching vibrations of both the hydroxyl –OH groups, N–H in amino groups, and stretching vibrations of aliphatic C–H [21]. Zeeshan et al., have demonstrated the response of the iron oxide NPs synthesized with extract from different parts of *Avicennia marina* dry fruit, seeds, leaves, and green fruit that were then evaluated for their antibacterial activity against *Xanthomonas campestris pv. vesicatoria*, *Erwinia spp.*, and *Escherichia coli* bacteria. Two grams of leaf extract nanoparticles resulted in 25 mm, 35 mm, and 45 mm zones of inhibition, respectively. All the nanoparticles subjected to X-ray Diffraction (XRD) showed diffraction peaks for the (220), (311), (222), (400), (422), (511), (440), and (622) planes at 29°, 35.5°, 36.8°, 43°, 55°, 57°, 62.8°, and 73°, which was a face-centered cubic structure with a decrease in crystallite size as atoms filled empty spaces left (7.1335 nm, 9.295 nm, 9.680 nm, and 10.128 nm), resulting in a surface area that is large and of high energy, and also the calculated hopping length gave the distance between magnetic ions, and here it was found that the lattice parameter increased with an increase in hopping length. The increasing size of the particle created two different effects on X-ray density that increased its value, but dislocation density decreased. The Williamson–Hall (W-H) plot’s slope was positive for all except leaf-employed synthesis, and the negative slope represented tensile strain and compressive strain. On the FTIR analysis, the presence of an extra functional group was confirmed due to the nature of the extract, and as nanoparticle size decreased, the bandgap (3.16 eV, 2.57 eV, 2.99 eV, and 2.52 eV) increased with an increase in bandgap kinetic energy and columbic interaction [2].Commonly called Rosella, i.e., *Hibiscus sabdariffa*, and used in herbal tea as an ingredient by Saod et al., in the biosynthesis of iron oxide NPs, its calyx’s extract with FeSO₄ solution was employed as a precursor, resulting in nanoparticles of spherical and coarse needle-like morphology on surface analysis, with a UV adsorption spectrum between 290 nm and 300 nm, and the atomic force microscope’s (AFM) three-dimensional image showed the folded mountain-like structure of metal bindings and oxide precipitation with an average grain size of 43.55 nm. The antibacterial activity of FeO NPs occurred by damaging proteins, nucleic acids, and cell membranes with reactive oxygen species like hydroxyl radicals, superoxide radicals, singlet oxygen, and hydrogen peroxide, leading to oxidative stress in cells. The nanoparticles as antimicrobial agents are able to produce ionic species through interaction with the active site and electrostatic interaction between the cell and the nanoparticle. The small-sized nanoparticles were effective, as they easily penetrated the membrane for interaction, and on analysis, FeO NPs combined with antibiotics increased their activity against bacteria [3].Bacterial strains like *Escherichia coli*, responsible for infections in the bloodstream and urinary tract; *Pseudomonas aeruginosa*, responsible for nosocomial infections and chronic wounds and urinary tract infections; and *Staphylococcus aureus*, which causes infections in the blood, were tested with iron oxide NPs by Yassin et al., which were synthesized with *Salvia officinalis* leaf extract as a reducing agent with ferric nitrate solution as precursors that got agglomerated into spherical morphology in which the particle distribution ranged from 10 to 80 nm. On UV analysis, the peaks at 200 nm and 349 nm corresponded to phytochemicals and surface plasmon resonance of nanoparticles, respectively, and EDX detected the presence of Fe and O with extra peaks for C and Si attributed to carbon tape and the effect of the capping factor in the extract. The zeta potential analysis concluded its hydrodynamic diameter to be 681.4 nm with a surface charge of -5.48 mV. The synergistic effect with tigecycline by the disk diffusion method showed 5%, 51.6%, and 38.85% of synergism for *Escherichia coli*,* Pseudomonas aeruginosa*, and *Staphylococcus aureus* [102]. In another study, Birusanti et al., observed effective antibacterial activity against *Salmonella enterica*, *Escherichia coli*,* Streptococcus agalactiae*, and *Staphylococcus aureus* by iron oxide NPs assisted with gooseberry leaf extract, which produced a diffraction pattern on XRD analysis for plane (1 1 0) at 27.489°, (2 2 0) at 31.749°, (4 2 2) at 45.4926°, (5 1 1) at 56.5786°, (6 2 2) at 66.5462°, and (5 3 3) at 75.5485° of cubic structure, with peaks at 3342.22 cm⁻¹, 2913.22 cm⁻¹, 2844.66 cm⁻¹, 1482.20 cm⁻¹, 1380.88 cm⁻¹, 1067.91 cm⁻¹, 872.95 cm⁻¹, 611.95 cm⁻¹, and 481.25 cm⁻¹ corresponding to OH stretching vibration for phenolic and alcoholic species; C–H stretching vibrations of the hydrocarbon chains; N–H stretching bands of aromatic amino groups and the C–N stretching bands of primary amines; Fe–O; out-of-plane bending of aromatic C–H stretching bonds; and C = O or C–O stretching vibrations on FTIR analysis. On Photoluminescence (PL) analysis, it showed excitation states for Fe (3d), O (2p), and Fe (4s) states that produce emission in the blue–violet region of the spectrum at 436 nm. On Thermogravimetric Analysis/Differential Thermal Analysis (TG/DTA) analysis, it showed mass loss at 93.2 °C of 5.96%, 195.5 °C of 16.4%, and 684.9 °C of 12.33% due to evaporation of adsorbed water and volatile substances, decomposition of organic groups, and finally, 49.05% of residual mass at 998.6 °C [52]. Rauf et al., used the plant extract of *Thevetia peruviana* assisted in the synthesis of iron oxide NPs and produced nanoparticles that showed spherical shapes with irregular surfaces upon surface analysis. The influence of biological metabolites through the presence of a bond on FTIR analysis belonging to phenolics, flavonoids, and proteins. The synthesised nanoparticles’ anticancer activity against it was very remarkable, with IC_50_ values of 44.09 ± 1.16, 65.09 ± 0.88 and 0.39 ± 0.10 against Hepatocellular carcinoma, lung carcinoma cell lines, and human ovarian carcinoma cell lines [141]. Similarly, iron NP synthesis with *Micromeria biflora* extract was obtained by Rauf et al., with a UV absorption peak for Surface plasmon resonance (SPR) of iron oxide NPs under analysis, cuboctahedral morphology and a peak that occurred for C, N, O and F under elemental analysis and further tested biological studies of anti-inflammatory action 90% with paw oedema on the third hour of treatment and anticancer tests against A498 cell line derived from renal tissue, HepG2 cell line derived from human hepatoma; NCI-H226 cell line derived from non-small cell lung tissue; and the 2780AD cell line derived from multidrug-resistant human ovarian cancer obtained 30.12 ± 0.34, 178.09 ± 0.87, 118.55 ± 0.43, 0.91 ± 0.24 [142].

The importance of extract efficiency was established by Islam et al., with zinc oxide NPs synthesized with blended onion waste peel at three different conditions for extract preparation. A using a microwave, B using mechanical shaking, and C using a hot plate in three different conditions. For A, B, and C, a varying quantity of deionized water with 50 g of blended onion waste peel (A1, B1, and C1 with 100 mL of deionized water; A2, B2, and C2 with 150 mL of deionized water; and A3, B3, and C3 with 200 mL of deionized water) was tested for its antioxidant activity with 150 mg of nanoparticle with 3.2 mL of a 100 µM DPPH sonicated solution, after which the absorbance was measured at 517 nm at different intervals to determine the optical density with the equation.

$$\:\mathrm{P}\mathrm{e}\mathrm{r}\mathrm{c}\mathrm{e}\mathrm{n}\mathrm{t}\mathrm{a}\mathrm{g}\mathrm{e}\:\mathrm{o}\mathrm{f}\:\mathrm{D}\mathrm{P}\mathrm{P}\mathrm{H}\:\mathrm{i}\mathrm{n}\mathrm{h}\mathrm{i}\mathrm{b}\mathrm{i}\mathrm{t}\mathrm{i}\mathrm{o}\mathrm{n}=\left\{\right(\mathrm{A}o-\mathrm{A}\mathrm{s})/\mathrm{A}o\}\times\:100$$

Where, A₀ is control and A_s_ is sample.

Resulted in intense peaks at 517 nm; the DPPH free radical scavenging activity was 63.85% and 71.53% at 45 min for A1 and C3, and it was found to be dose and size dependent. C3 resulted in an effective zone of inhibition as a result of size dependency on antibacterial activity using the disc diffusion method against *Staphylococcus aureus*,* Escherichia coli*, and *Pseudomonas aeruginosa* as 12.00 ± 1.52 mm and 13.50 ± 0.76 mm, 8.83 ± 0.54 mm and 13.17 ± 0.82 mm, and 13.00 ± 1.00 mm and 22.00 ± 0.58 for A1 and C3. This was the result of increased surface area that increased diffusion by increasing surface oxygen species that destroy the membrane responsible for death or rupturing the cell wall by contact that releases free ions, disintegrating the cell’s carbohydrate and protein. The synthesized samples were tested with UV, revealing A1 and C1 to be effective through peaks at 322 nm and 318 nm with optical band gaps of 3.47 and 3.48 eV. Both round and hexagonal-shaped nanoparticles for A1 and C3 existed; here, the change in shape was attributed to an increase in the concentration of extract, with average crystallite sizes of 72.60 nm and 57.38 nm, respectively, for A1 and C3 [25]. Badri et al., revealed the green synthesis mechanism of ZnO NPs with *Chenopodium exsuccum* plant extract, with flavonoids, phenolics, and alkaloids stabilized and also reduced Zn ions; then they nucleated to form nanoparticles. It generated free radicals that were scavenged by the antioxidant nature of the extract. Then it was further tested on its antioxidant activity by DPPH scavenging activity, resulting in an IC_50_ value of 23.1 µg/mL for ZnO, and then on antibacterial activity against *Escherichia coli*,* Micrococcus luteus*,* Staphylococcus aureus*, and *Klebsiella pneumoniae*, it exposed the inhibited zones at concentrations of 4 µg/mL as 12.9 ± 0.13, 4 µg/mL as 16.9 ± 1.6, 4 µg/mL as 11.2 ± 0.18, and 2 µg/mL as 12.8 ± 0.5, and its MIC test with 100 µg/mL of ZnO NPs resulted in 83%, 81%, 67.3%, 57.6%, and 39.6% bacterial growth inhibition in 24 h. This ZnO NP characterization disclosed its average crystallite size using Scherrer’s equation as 21 nm of inhomogeneous, elongated-shaped nanoparticles with a band gap of 3.46 eV and an absorption peak at 370 nm. The formation of metal oxide nanoparticles was confirmed with a peak at 481 cm⁻¹ on FTIR analysis. Also, EDX unveiled the presence of Zn and O along with Si, P, and S due to the usage of plant extract, and X-ray Photoelectron Spectroscopy (XPS) analysis peaks denoted the existence of O 1s at 531 eV, C 1s at 286 eV, Zn 3d at 11 eV, Zn 3p at 89 eV, Zn 3s at 141 eV, Zn LMM at 499 eV, Zn 2p1/2 at 1022 eV, and Zn 2p3/2 at 1045 eV [46]. The leaf extract of *Syzygium cumini* with ZnO produced a wurtzite structure of 35 nm crystallite size on XRD analysis by Yada et al., with peaks at 2900–3600 cm⁻¹, 1585 cm⁻¹, 1398 cm⁻¹, 1332 cm⁻¹, 1017 cm⁻¹, and 933 cm⁻¹, due to O–H stretching vibrations, C–H bonds of alkanes, C–C stretches, N–H bonds of amides and amines, and C–N bonds of aliphatic amines and aromatic ring stretches; and at 667, 614, and 504 cm⁻¹, it’s due to Zn–O with stretching vibrations on FTIR analysis. On UV-visible analysis, peaks at 373 and 294 nm corresponded to ZnO and aromatic compounds in the extract. The cytotoxicity study with the HepG2 cell line using the MTT assay increased concentration and showed a decrease in cell viability. The antimicrobial activity against 100 µl of *Aspergillus niger* and *Pseudomonas aeruginosa* showed 6.33, 6.33, 5.66, 6.0, and 11.66 mm and 7.3, 7.6, 7.3, 9.3, and 11.66 mm of zone of inhibition with 50, 125, 250, 500, and 1000 µg from the disk diffusion method and the Kirby-Bauer method, respectively. The MIC test was carried out with McFarland standard dilutions with *Aspergillus niger* and *Pseudomonas aeruginosa* was found to be 8 µg/ml and 0.1 µg/ml, respectively. The antioxidant activity, by employing the DPPH scavenging assay, obtained an IC_50_ value of 10.54 ± 1.49 µg/ml, and on the anticorrosive test, the value of CIE was 90.01% with 100 ppm of ZnO, and its antifungal activity with 50, 125, 250, 500, and 1000 µg/disc by the disk diffusion method with potato-dextrose-agar (PDA) and 100 µl of *Aspergillus niger* fungal culture acquired 6.33, 6.33, 5.66, 6.0, and11.66 mm sized zones [49].

#### Plant extract-derived metal oxide doped nanoparticles

Sarani et al., produced Bi₂O₃, Ag-doped Bi₂O₃, and Cu-doped Bi₂O₃ NPs with *Biebersteinia multifida* extract as a reducing agent, and their formation was confirmed through EDAX with the presence of the respective elements. The particles were of platelike morphology with the monoclinic structure of bismuth oxide with the absence of diffraction peaks for Ag and Cu on XRD analysis, but the change in intensity of peaks indicated a change in particle size when doped, whereas Raman analysis showed the existence of peaks at 83, 119, 139, 153, 184, 220, 281, 305, 459, 514, and 598 cm⁻¹. Then it underwent a cytotoxicity test in medium cultured with U87 and 3T3 cell lines and nanoparticles in a range of 0 to 2560 µg mL⁻¹, which was incubated and later treated with MTT solution (5 mg mL⁻¹). It increased cell viability for copper-doped bismuth NPs, where copper produced stress by oxidation that led to malfunction of mitochondria and death. The other two nanoparticles showed cell viability, and they showed morphological changes on microscopical imaging. On the minimum inhibitory concentration test, no colour change was observed at the lowest concentration, indicating inhibition of bacterial growth. By the microdilution method, *Pseudomonas aeruginosa* and *Staphylococcus aureus* bacteria that were cultured on nutrient broth were mixed with 0 to 5120 µg mL⁻¹ of nanoparticle concentration with 105 colony-forming units, incubated, and added to Triphenyltetrazolium chloride (TTC) solution (0.5 mg mL⁻¹); then the colour changed from pale yellow to pink, which indicated the MIC value, and resulted in no MIC being observed for all three samples, but this doesn’t conclude it will be ineffective, as it depends on various conditions. To monitor biofilm activity by *Pseudomonas aeruginosa* and *Staphylococcus aureus* in 10^5^ colony-forming units from Tryptic Soy Broth with nanoparticles of concentration 0 to 5120 µg/mL⁻¹ that were incubated, the medium was removed, washed, and fixed using heat, in which biofilm was stained with crystal violet and dissolved in acetic acid, and then finally absorbance was measured that reflected the concentration-dependent activity as it increased active site number on both Ag and Cu doping but was very effective on Cu doping, as the inhibition by the doping elements depends on contact with bacterial cells that released ions that ruptured cell membranes and disrupted cellular functions [1].

Shkir et al., prepared MgO and Ag-doped MgO NPs with the plant extract of *Morinda tinctoria* for the investigation of antimicrobial activity. The major activity was unveiled by Ag-doped MgO against Staphylococcus aureus and *Escherichia coli*, which was due to ROS (Reactive Oxygen Species) generation that kills pathogens by damaging their membranes, developing craters, and shrinking the cells that leak the cell’s contents. On Ag integration, the changes identified were 70% to 88% on crystallinity, 64% to 76% on porosity, and 3850 nm² to 5896 nm² on surface area for Ag/MgO and MgO. Then in XRD, the average crystallite size was 28 nm to 21 nm for MgO and Ag/MgO. In Ultraviolet–Visible Diffuse Reflectance Spectroscopy (UV-DRS), decreased absorbance was seen as a decrease in absorbance from UV to visible with a decrease in bandgap from 4.68 eV to 3.18 eV, exhibiting increased O on EDAX analysis. In DLS (Dynamic Light Scattering), MgO’s large grain size was exhibited with dispersion values of 0.634 and 0.43. In zeta potential, MgO had a value of -20 eV and Ag/MgO had a value of 16.32 eV; this increase was due to increased electrostatic force repulsion and decreases in oxygen defects that reduced agglomeration. In an XPS study, a spectrum for Ag, Mg, O, and C occurred from Ag/MgO NPs [54].

Tiki et al., carried out the production of silver-doped metal oxide NPs whose microbial efficacy from literature was by death of cells when Ag⁺ interaction created reactive oxygen species or silver’s ability to damage mitochondria that caused a lack of energy, and here various concentrations of silver Ag₁₋ₓAg₁₋ₓNO₃ (x = 0.2, 0.4, 0.6, 0.8, and 1.0) doped with SnO₂ were synthesized with root extract of *Zingiber officinale*, getting Ag-doped SnO₂, where the extract reduces Ag⁺ and Sn⁴⁺ and stabilizes by donating electrons through coprecipitation before which the extract was subjected to qualitative phytochemical analysis confirmed the presence of alkaloids, tannins, phenols, flavonoids, terpenoids, and glycosides, and the doping was confirmed through elemental mapping. On XRD analysis, Ag doping broadened the peak with a decrease in particle size and generated defects with a decrease in average crystallite size on increasing dopant. The FTIR analysis showed the peak at 622.239 cm⁻¹ corresponded to Ag − O−Sn bonding of the Ag-doped SnO₂ nanoparticles, and a 300–800 nm absorption peak was seen in the UV analysis. Increased Ag doping resulted in a redshift shift towards higher frequency with a decrease in band gap values of 3.57, 3.41, 3.29, 3.19, and 3.08 eV for pure SnO₂, 0.2 Ag-doped SnO₂, 0.4 Ag-doped SnO₂, 0.6 Ag-doped SnO₂, and 0.8 Ag-doped SnO₂ NPs, owing to electron transition energy. The antibacterial test resulted in the highest activity for 0.8 Ag SnO₂ on gram-negative bacteria that are of small crystallite size, with increased surface area contact with microbes, released Ag⁺ that interacted with the cell membrane for entry, and then damaged DNA and finally caused death [2].

Muthuraj et al., fabricated nanoparticles with *Boerhavia diffusa* leaf-extract-mediated (BDE) and got magnesium-doped-nickel oxide NPs (Mg-doped NiO-BDE) and disclosed distorted spherical-shaped nanoparticles of 51–55 nm in size, and their formation was confirmed with FTIR, EDX, and XPS analysis that was examined for antibacterial activity of nanoparticles of undoped NiO, leaf extract of BDE, and different concentrations of Mg (10, 30, 50 mol%) in MgNiO of 10, 30, 50 mg, with positive control Streptomycin disc against *Escherichia coli* and *Staphylococcus aureus* and revealed that activity for 50 mg concentration was 18 mm and 11 mm, 13 mm and 12 mm, 11 mm and 12 mm, 12 mm and 12 mm, and 14 mm and 14 mm zone of inhibition for extract. The undoped and Mg-doped (10, 30, 50 mol%) NiO NPs and deduced the following as possible mechanisms: nickel replacement of metalloenzymes resulted in reduction of enzyme activity by binding to non-metalloenzymes, binding to enzymes that affected its activity, and generating free radicals on the cell membrane that led to dysfunction and cell death. Then it was further subjected to antifungal activity against *Candida albicans* and *Aspergillus niger* on 50% MgNiO with 50 mg, as it showed effective activity on the antibacterial test with a positive control (20 µg clotrimazole), exhibiting 10 mm of inhibition against *Candida albicans* after 24 h and 29 mm and 25 mm of inhibition against *Aspergillus niger* after 24 h and 48 h, respectively, by the agar diffusion method and the food poisoning method. *Aspergillus niger*’s maximum inhibition was only 27%, which dropped on day 5, whereas *Candida albicans*’s was 58% and dropped on day 5. Antioxidant activity revealed the concentration-dependent scavenging activity of 15.2%, 38.6%, 48.7%, 57.2%, and 65.5% when exposed to concentrations of 20, 40, 80, 200, and 400 µg/mL of 50 mol%. Mg-doped NiO NPs in Mg have increased the production ability of radical oxygen species. The antidiabetic activity was found by testing the inhibition of alpha-amylase as 28.2%, 46.3%, 57.7%, 76.5%, and 87.1% inhibition on Mg-doped NiO-BDE (concentrations = 20, 40, 80, 200, and 400 µg/mL) due to the flavonoid-phytochemical’s nature. The anti-inflammatory was by bovine serum albumin (BSA) denaturation assay, showing a 4–15% increased inhibition compared to NiO and stabilizing the role of Mg. The cytotoxicity test with A549 cell density resulted in its cell viability as 94.8 ± 0.2%, 89.4 ± 0.8%, 75.1 ± 0.9%, 35.7 ± 0.7%, and 14.3 ± 1.0% for 25, 50, 100, 250, and 500 µg/mL of the Mg-doped NiO-BDE, and IC_50_ was 185.6 µg/mL, whereas for NiO it was 50 µg/mL, and finally, tested on in vitro wound healing—wound closure by scratch using 100 µg of 50 mol% Mg-doped NiO-BDE with an L929 cell line revealed its activity was lesser than the collagen-synthetic polypeptide blend [75].

Al-Momani et al., studied skin pathogens, *Propionibacterium acnes*, a common bacteria present in human skin that is generally treated with antibiotics, and started to show antibiotic resistance; hence, they were expecting an effective therapy by nanoparticles, so they used Ag and ZnO NPs synthesized with seed extract of *Peganum harmala*, which acted as both a reducing and capping agent, that is, with a phenolic content of 8.11 ± 1.7 mg GAE/100 mg and a flavonoid content of 0.76 ± 0.12 mg QE/100 mg extract from the Folin–Ciocalteu method, and the extracts’ phenolic and flavonoid content played the role of single oxygen quenchers, hydrogen donors, and chelating metal, reducing metal ions with therapeutic values. The agar well method against 1.5 × 10⁸ CFU/mL of *Propionibacterium acnes* in two strains was employed in Mueller–Hinton agar plates and showed the mean inhibition zone to be 11.0 ± 1.0, 9.7 ± 1.2 mm, and 9.7 ± 0.6 mm with 250 µg/mL as the MIC (Minimum Inhibitory Concentration) and MBC (Minimum Bactericidal Concentration) value for ZnO, whereas the Ag NPs zone of inhibition was higher than ZnO at 13.2, 11.7, and 11.0 mm. The synthesized nanoparticles of silver and zinc oxide had an average particle size of 10 nm and 50 nm, high crystallinity, and purity, as shown by the diffraction peak with a negative charge on the surface of -34.7 mV. The biofilm inhibitory effect on crystal violet-stained *Propionibacterium acnes* by the spreading method was found to be 62.5 µg/mL and 3.9 µg/mL by the creation of cell lysis, and ≥ 15.6 µg/mL and > 31.25 µg/mL were the concentrations reducing the biofilm for Ag NPs and ZnO NPs [40]. Similarly, Zn doped with Bb led to Bb-ZnO, which was synthesised by the coprecipitation method with *Barleria buxifolia* leaf extract by Alalawy et al., and got a spherical shape on surface morphology whose size ranged from 2 to 50 nm. Its antioxidant activity with DPPH, ABTS (2,2′-azino-bis (3-ethylbenzothiazoline-6-sulfonic acid), and Nitric oxide scavenging assays at different dosage 0, 40, 60, 80,100 and 120 µg/mL of Bb-ZnO and ascorbic acid were measured with Ultraviolet–Visible Spectroscopy (UV–VIS) spectrophotometer at three different wavelength 515, 734, 546 nm for DPPH, ABTS and nitric oxide revealed 62 µg /mL and 54 µg /mL, 67 µg /mL, and 58 µg /mL and 92 µg/mL and 77 µg/mL to be IC_50_ values of Bb-ZnO NPs and L-ascorbic acid and the cytotoxicity activity by MTT assay 5 mg/ml with lung cancer cells A549 and Bb-ZnO − 20–120 µg /mL resulted in IC_50_ values to be 67.2 µg /mL whereas Cisplatin’s value is 43.5 µg /mL, on twin staining analysis the apoptosis using acridine orange and ethidium bromide staining, and 4′,6-Diamidino-2-phenylindole dihydrochloride (DAPI) found the cellular damage and nuclear condensation produced 29.36 ± 1.44 and 43.25 ± 1.94, and 29.36 ± 1.44 and 43.25 ± 1.94 mm zones, for Bb- ZnO and coisplatin, the ROS production assays by employing DCFH-DA (2′,7′-Dichlorodihydrofluorescein Diacetate) as an oxidation-sensitive fluorescent ROS marker in A549 viable cells resulted in emission of fluorescence colour and distorted morphology for ZnO and cisplatin, then on mitochondrial transmembrane potential measurement employed Rhodamine 123 with A549 cell and on examination with fluorescence microscopy found ZnO caused distortion in mitochondria and the antibacterial activity by agar well diffusion method against gram-negative (*Escherichia coli*,* Pseudomonas aeruginosa* ) and gram-positive (*Staphylococcus aureus*) at various concentrations 25 µg/ml 50 µg/ml 75 µg/ml 100 µg/ml produced effective activity with increase in concentration [44].

The phytochemical analysis was conducted by Muhammed et al., with the extract of *Lupinus albus* with flavonoid as the major component that reduced metal ions and stabilized and also indicated presence of tannins, alkoids, phenolic and glycoside compound so was employed in synthesis of ZnO and Mg-ZnO NPs that showed inhibition zones for nanoparticles to be 24 mm, 22 mm, 21 mm, 21 mm and 15 mm, 21 mm, 19 mm, 17 mm, 18 mm, and 13 mm against *Escherichia coli*,* Bacillus cereus*,* Salmonella typhi*, and *Staphylococcus aureus*, one fungal strain *Candida albicans* for Mg-ZnO and ZnO by disc diffusion method with 200 mg/mL of synthesised sample by electrostatic interaction that destroyed the membrane permeability between cell membrane and ions or by absorbing light the nanoparticle creates holes in valence band that split H_2_O to into OH^−^ and H^+^ on jump of electron to conduction band that reacts with dissolved oxygen to forms O_2_^•−^, then H^+^ to generate the HO_2_^•^ radical reacted to form Hydrogen peroxide that damaged membrane of cell by its oxidizing nature ZnO and Mg-ZnO NPs with average crystallite sizes calculated from the Debye−Scherrer formula were 28.1 and 34.4 nm, with absorbances peak at 357 and 363 nm, and band gaps are 3.25 and 3.02 eV by Tauc relationship from UV-vis analysis [51].

The plant extract was prepared with *Withania* as a reducing and capping agent by Hasan et al., for synthesising Mn-doped ZnO NPs in different ratios of Zn and Mn (1:1, 1:2, 4:1, and 2:1). It showed visible morphological changes on changing ratio and size, and the 11–14 nm amorphous tetragonal structural morphology was assessed for antibacterial activity on *Escherichia coli* and *Staphylococcus aureus* by the disc diffusion method with various concentrations of composites, such as 10, 20, and 40 µg/mL. Effective activity was observed with increasing concentration of Mn due to the negative nature of the bacterial cell’s interaction with the nanocomposite, which produced reactive oxygen species that destroyed cells. The synthesised nanocomposite with a 2:1 ratio showed the highest absorption band at 350 nm on UV analysis with band gap values of 3.9 eV, 3.7 eV, 3.4 eV, and 3.3 eV for 4:1, 1:1, 2:1, and 1:2 calculated with Tauc equations αhυ = A(hυ − Eg)ⁿ, whereas others did not show peaks, indicating the absence of the reducing agent’s role, and further on EDAX analysis, the presence of zinc, manganese, carbon, oxygen, sodium, calcium, carbon, and hydrogen was detected, indicating the use of biological material as a reducing agent [20].The bandgap’s role in antioxidant activity was revealed by Rana et al., on a DPPH free radical assay on both green-synthesised zinc oxide (ZO) and yttrium-doped ZO NPs (YDZO) from *Murraya koenigii* extract with crystallite sizes of 36.15 and 36.369 nm and bandgaps of 3.155 eV for ZO and 3.136 eV for YDZO nanoparticles, as the bandgap showed variation with crystallite size with spherical morphology with the DPPH scavenging assay and ascorbic acid as a reference on varying concentrations from 25 to 800 µL, and the calculated scavenging rate was higher for ascorbic acid (88.498 > ZO 80.359 > YZO 76.127) at 800 µL. Antibacterial potential by the well diffusion method against both *Bacillus subtilis* and *Escherichia coli* bacteria gave the highest zone of inhibition for 20 mg/m⁻¹ as 32 and 34 mm and 29 and 34 mm, respectively. Through this, it was concluded that higher concentrations of ZO are more effective for both gram-positive and gram-negative bacteria [74].

The various concentrations of double doping of ZnO with Al and Fe (ZnO 0.048, Fe 0.001, Al 0.001; ZnO 0.046, Fe 0.001, Al 0.003; and ZnO 0.044, Fe 0.001, Al 0.005) with *Murraya koenigii* leaf extract were experimented on by Verma et al., and it resulted in a hexagonal wurtzite structure with no additional peaks other than those for Fe and Al, with a decrease in crystallite size from 31 to 17 nm, microstrain from 1.69 × 10⁻³ to 1 × 10⁻³, and band gap from 2.99 to 2.88 eV, and an increase in volume from 47.33 to 47.50 on increasing the concentration of Al. The clustered spherical and irregular surface morphology on concentrated ZnO with Al and Fe NPs with elemental composition confirmed the presence of peaks for Zn, Fe, Al, and O in the EDAX spectrum with a decrease in average particle size from 34 to 20 nm on Transmission Electron Microscopy (TEM) analysis. The antibacterial activity was observed at various concentrations (5, 15, and 25 mg/ml) by the well diffusion technique against *Escherichia coli*,* Bacillus subtilis*, and *Staphylococcus aureus* and found that the increased activity was with an increase in concentration and a decrease in size. The antimicrobial activity followed three steps: generation of reactive oxygen species, reduction, and then damaging the cell wall and antioxidant activity. Then it was evaluated for DPPH assay, which illustrated an absorption peak at 517 nm with effective activity on increasing concentrations for ZnO 0.044, Fe 0.001, and Al 0.005 from 25 to 800 µg/ml as the DPPH assay increased the surface-to-volume ratio [50].

#### Plant extract-derived metal oxide nanocomposites

Ag@Sm₂O₃/ Reduced Graphene Oxide (rGO) synthesized with *Punica granatum* peel extract produced nanoparticles sized 10–20 nm with a pore size of 18.1 nm and a surface area of 97.2 m²/g by the process of first preparing a GO (Graphene Oxide) suspension solution, then adding copper nitrate, silver nitrate, and extract to a stirrer with NaOH to bring the pH to 9, and then cooking with reflux conditions followed by separation using a magnetic field and oven drying. The study on elemental composition divulged peaks at 374.2 and 367.9 eV belonging to Ag 3d3/2 and Ag 3d5/2, 1110.5 and 1083.3 eV for Sm 3d3/2 and Sm 3d5/2, and 532.1 eV for the O 1s peak. Then the antimicrobial study against *Escherichia coli* didn’t show effective activity, whereas undoped Sm₂O₃ exhibited an inhibition zone of value 11.23 ± 0.42 mm, as reported by Mahalakshmi et al., [115]. A 9–16 nm sized nanocomposite of Ag-doped ZnO/MgO was synthesized by utilizing *Mentha pulegium* plant extract on the sol-gel method by Narm et al., Initially, an extract of *Mentha pulegium* plant extract was obtained and stirred with a salt solution of 7.43 g of Zn(NO₃)₃0.6 H₂O, 6.41 g of Mg(NO₃)₃0.6 H₂O, and 0.042 g of Ag(NO₃)₃ at 75 °C and then was calcinated at different conditions: 400, 500, and 600 °C. These variations with temperature resulted in changes in various factors about grain size (about 27.3, 27.8, and 28.2 nm), crystallinity (62%, 65%, and 83%), and surface area (154, 152, and 149 m²), with band gaps of 3.58, 4.58, and 4.78 eV. Then, tested with the CT-26 cancer cell line by MTT test with 0, 125, 250, 500, and 1000 µg/mL concentrations, the synthesized nanocomposite showed its death in 24 h with an IC_50_ value of about 250 µg/mL [143].

The synergistic interaction of plant extract with metal oxide nanoparticles has emerged as an inexpensive, rapid, reliable approach. Based on the reviewed studies, the phytochemicals phenolics, flavonoids, tannins, and alkaloids were identified to induce nanoparticle formation. Zinc oxide, copper oxide, and iron oxide nanoparticles were explored most in the investigation of metal oxides via plant extract as antimicrobial agents. These demonstrated enhanced actions are owed to the collective properties of smaller crystallite sizes, metal precursors, and phytochemical composition [97, 110, 131]. Among the adopted modification strategies, incorporating silver into metal oxide nanoparticles using plant extracts is reported to improve antimicrobial performance by ion release and production of ROS [2, 40, 54,115 ].

### Microbe-mediated biosynthesis of metal oxide nanoparticles

Fabrication of nanoparticles using living organisms such as bacteria, algae, fungi, yeast, and viruses, or using the extracted biomolecules (polysaccharides, DNA, or proteins), is referred to as microbial nanotechnology [31, 47, 48, 144]. This field combines nanotechnology and microbial technology [42] and has gained favorable attention for the synthesis of metal oxide nanoparticles, with microbes as a viable alternative [33, 35]. These nanoparticles find applications in different fields, including agriculture, the food industry, the environment, biomedicine, and electronics. In the agricultural aspect, environmental changes have dramatically impacted crop production globally over the past decade [37, 47]. Microbes can accumulate and tolerate metal. There are two routes of reduction, extracellular and intercellular, in a supernatant. The supernatant is obtained by growing microbes in a suitable medium, followed by centrifuging the broth containing the microbial cells to isolate the supernatant, which contains a range of enzymes that interact with metal ions to produce ions in lower valency states [31, 33, 35, 57]. The property of resistance to heavy metals in microorganisms is due to their inbuilt chemical detoxifying and ion efflux nature [42].

The single microorganism can be involved in synthesizing both the methods that are determined by environmental conditions [48]. The mechanism of microbial detoxification in intracellular synthesis involves the absorption of metal ions, which are then transformed by enzymes into nanoparticles within the cells; these nanoparticles exhibit novel physicochemical properties but are limited by factors such as substrate concentration, exposure time, pH, and temperature. In contrast, extracellular synthesis occurs when metal ions are absorbed on the cell surface, where they interact with the cell’s enzymes to form nanoclusters of nanoparticles that diffuse from the cell wall. It is simple to harvest, fast to produce, and straightforward to purify for large-scale production [31, 35, 42, 47, 48, 57]. The extracellular is preferred widely, as it has facile separation, whereas the intracellular involves ultrasonication and centrifugation for biomass removal, where the cell wall is broken for the recovery of nanoparticles [42, 57]. The mechanism of reduction by biomolecules alters the nanoparticle’s physical characteristics and chemical content [42]. The reduction and stable dispersion take place with the assistance of enzymes such as reductase, laccase, amino acids, polyhydroxyalkanoates, sulfur-containing proteins, nitrate reductase, polysaccharides, peptides, catalase, cytochrome reductase, and ligninase [32, 42, 48,145 ]. The size of the particle and its monodispersity were supported by the type, strain, and growth medium of the microorganism; the substrate’s concentration; the temperature; the pH; and the time taken for the reaction [7].

#### Microbe-derived metal oxide doped nanoparticles

##### Bacteria-mediated metal oxide nanoparticles

The biomass filtrate from *Lactobacillus rhamnosus* was employed by Abdel-Maksoud et al., for preparing TiO_2_ NPs with inoculated strain de Man, Rogosa, and Sharpe broth (MRS) then incubating at 35 ± 2 °C for 24, then centrifuging for 10 min at 1000 rpm followed by rinsing that was then suspended in 100 mL of dis. H_2_O with incubation for 24 h and then finally was centrifuged to collect supernatant that was mixed with [TiOCH(CH_3_)_2_]_4_ with stirring for one hour on addition of NaOH to attain pH 8 and precipitate formed was then incubated, washed with water and dried in oven for 3 h at 200 °C produced nanoparticles of 7.5 nm with spherical shape of average particle size 15 nm, anatase form was confirmed through circular diffraction ring pattern in SAED (Selected Area Electron Diffraction) and two negative values – 19.9mV and – 36.8 mV on zeta potential indicated increased stability the repulsion between them prevented agglomeration then analysed for its biocompatibility with MTT method 100 µL of normal cell lines WI38 (human fibroblast lung tissue) and HFB4 (human normal melanocytes) with 50 µL of MTT solution and using ELIZA reader to measure absorbance at 570 nm exhibited the dose dependent nature and found its safe on (≤ 300 µg mL^–1^) IC_50_ the concentration of nanoparticle inhibited 50% of cell viability values were 341.9 ± 12.8 and 368.9 ± 1.6 µg mL^–1^ for WI38 and HFB4 and its antifungal activity with varying concentration found to be dose dependent with zone of inhibition for *Aspergillus flavus ED1*, *Aspergillus versicolor ED3*, *Penicillium citrinumED4*, *Actinidia chinensis ED8*, *Aspergillus ustusED11*, and *Penicillium chrysogenum ED14* as 17.7 ± 0.6 mm, 20.3 ± 1.5 mm, 18.7 ± 0.6 mm, 20.3 ± 0.5, 18.3 ± 0.6 mm and 17.7 ± 1.2 mm [146].

The bacterial strain pp6, non-motile, short-rod bacterial isolates with glossy colonies on solid agar media were employed by Ishwarya et al., to synthesize ZnO NPs that showed 24.33 ± 0.56 mm, 22.16 ± 0.48 mm, 20.52 ± 0.89 mm, 18.74 ± 0.89 mm, and 16.09 ± 0.75 mm zones of inhibition against *Enterobacter aerogenes*,* Enterococcus faecalis*,* Serratia marcescens*,* Streptococcus pyogenes*, and *Staphylococcus aureus* by the agar well technique, while the antibiotic ciprofloxacin’s zone of inhibition ranged between 24 and 27 mm. The 15 µg of ZnO exhibited its activity by decreasing the biofilm formation against *E. coli* and *Streptococcus pyogenes*, and then the cytotoxicity analysis by MTT test with HT-29 cells showed cell viability of 26.54 ± 0.48% for 80 µg. The ZnO NPs were in a hexagonal zincite structure with diffraction peaks at 33.62°, 59.34°, and 69.92° that corresponded to (002), (102), and (004) planes, with an absorbance pattern at 300.90 nm on UV-visible spectra with an average diameter ranging between 230 and 280 nm [37].

##### Fungi-mediated metal oxide nanoparticles

The synthesis of Co₃O₄ and ZnO NPs was carried out by Omran et al., with the assistance of the fungus *Aspergillus* species, to determine their antibacterial activity based on the effect of the dispersion medium and also based on the morphology of the synthesized nanoparticles, in which Co₃O₄ was of a flaked-shape structure and ZnO was of poly-shaped nanoclusters that comprised quasi-spherical and oval-shaped particles that are of high stability with zeta potential values of + 26.5 mV and + 22.4 mV. Co₃O₄ and ZnO NPs (5.0 mg/mL) dispersed in ethylene glycol against foodborne pathogens *Escherichia coli* and *Salmonella enterica Serovar Typhimurium*, and *Staphylococcus aureus* in comparison with streptomycin (1 mg/mL) by agar well diffusion assay showed efficacy on metal oxide dissolved in ethonal and proved its stablility, its MIC and MBC values were monitored using broth microdilution assay by varying its concentration from 0.078 to 10 mg/mL revealed MICs of Co_3_O_4_ against *Escherichia coli*,* Salmonella enterica Serovar Typhimurium*, and *Staphylococcus aureus* were 2.5, 2.5, and 1.25 mg/mL, MICs of ZnO NPs were 2.5 mg/ mL for all and MBCs of Co_3_O_4_ nanoparticles against *Escherichia coli*,* Salmonella enterica Serovar Typhimurium*, and *Staphylococcus aureus* were 5, 5, and 2.5 mg/mL, MBCs of ZnO nanoparticles were 5 mg/mL for all against the three tested foodborne bacterial pathogens respectively and using Field Emission Scanning Electron Microscopy (FESEM) observed its morphology during activity with pathogen and after treatment as its it already proved that other than spherical shaped particles showed increased mortality due to membrane damage, distortion, protrusions, cell debris generation of pit, twisting, shrinkage of cells and even nanoparticles deposition was observed and revealed the mechanism a electrostatic force between positively charged Co ions and negatively charged bacterial cell resulted in cell wall degeneration whereas ZnO penetrates on gram positive ones while diffuse on gram negative ones then generated reactive oxygen species, discharged metal ions, that caused oxidative stress by inhibiting the growth and finally death [57].Cobalt oxide NPs were synthesised with various extracts of *Fusarium oxysporum* and *Bacillus subtilis* from methanolic and aqueous extracts of *Aerva javanica*, as documented by Mubraiz et al., This resulted in average particle sizes of 33.4 nm, 31.2 nm, 68.9 nm, and 39.23 nm. Its investigation against *Bacillus subtilis*,* Staphylococcus aureus*,* Escherichia coli*, and *Pseudomonas aeruginosa* obtained an MIC for *Bacillus subtilis* with synthesised cobalt oxide NPs of 1.6 mg/mL and for *Fusarium oxysporum* with synthesised cobalt oxide MIC against *Bacillus subtilis* and *Escherichia coli*,* Staphylococcus aureus* and *Pseudomonas putida* of 3.13 mg/mL and 1.6 mg/mL, respectively. Cobalt oxide from methanolic extracts of *Aerva javanica* ranged from 6.25 to 3.13 mg/mL, and cobalt oxide from aqueous extracts of *Aerva javanica* against *Bacillus subtilis*,* Staphylococcus aureus*,* Escherichia coli*, and *Pseudomonas aeruginosa* was 6.25 mg/mL, 1.6 mg/mL, 12.5 mg/mL, and 3.13 mg/mL. The cobalt oxide NPs from *Fusarium oxysporum* extract and *Bacillus subtilis* exhibited effective antifungal activity with inhibition of 25.3% and 20% against *Fusarium moniliforme* and *Fusarium solani* [59].Cell filtrate of the fungus *Fusarium chlamydosporum* isolated from the leaves of the *Eucalyptus sideroxyl* on plant and 0.1 M of ZnSO₄ were used in the synthesis of ZnO by Hammad et al., resulting in 64.61% of Zn and 35.39% of O with spectra at 1.5, 8.5, and 9.6 MeV for Zn and 0.9 to 1.1 MeV for O; the absorption peaks at 320 nm under UV-Vis spectra; a hydrodynamic diameter of 15.77 nm; a polydispersity index of 0.952; and a zeta potential value of 8.01 with spherical and hexagonal shapes internally. It was subjected to antibacterial analysis against *Pseudomonas aeruginosa* and *Escherichia coli*, getting its MIC to be 1.95 µg/mL and 0.98 µg/mL, whereas in anticancer activity, when monitoring least inhibitory concentrations against HCT-116 and CACO2 cell lines, with concentrations of 5.06 µg/mL and 6.07 µg/mL, its cell viability was dose-dependent against CACO2 cells with concentrations of 0.0, 0.05, 0.1, 0.2, 0.39, 0.78, 1.56, 3.125, 6.25, 12.5, 25, 50, and 100 µg/mL of ZnO, showing 100, 100, 100, 100, 96.03, 87.24, 70.81, 54.12, 32.39, 21.4, 10.81, 6.53, and 2.74% cell viability [16].

Antibacterial activities of ZnO NPs were observed by Sharm et al., synthesized from the white-rot fungus *Phanerochaete chrysosporium* by the process of coprecipitation with precursors of extract, glycerol, ZnSO₄0.7 H₂O, and NaOH. It obtained nanoparticles as antibacterial agents by employing the agar well diffusion method against *Escherichia coli* and *Staphylococcus aureus* with wells of 7 mm in diameter at varying concentrations (from 0.09 lg/mL to 200 lg/mL). ZnO obtained a zone of inhibition in the range of 18–22 mm and 11–23 mm with properties like a hexagonal shape, an average size of 83.9 nm to 94.2 nm, and a bandgap of 3.01 eV with peaks at 3848–3618 cm⁻¹, 531 cm⁻¹, and 2359 cm⁻¹ on FTIR analysis, whereas it showed peaks at 388 nm, 403 nm, and 428 nm on UV–Vis analysis due to ZnO’s photoluminescence and oxygen vacancy. On TG analysis, weight loss occurred at 224 °C and between 224 °C and 400 °C due to evaporation of 77 molecules of surface-adsorbed water, and the other was possibly due to elimination of hydroxyl groups [145]. The sol-gel process was adopted by Ganesa et al., in the synthesis process of ZnO NPs assisted with endophytic fungi *Periconium sp*. Obtained particles with an average crystallite size of about 40 nm with a hexagonal wurtzite structure with a value of − 88.6 mV on zeta potential, hydrodynamic particle size of 338 nm, and band gap energy of 3.15 eV and quasi-spherical shape internally showed their antioxidant activity to be 85.52% with 100 µg/ml, and antimicrobial activity against *Staphylococcus aureus*,* Escherichia coli*, and *Candida albicans* zone of inhibition were about 16, 14, and 24 mm on using the agar well diffusion method with a concentration (150 µl) of ZnO synthesized with half maximal inhibitory concentration as 22, 38, and 21 µg/ml, and their antioxidant activity on employing radical scavenging activity at concentrations 20, 40, 60, 80, and 100 µg/ml got 78.78 ± 0.3, 81.08 ± 1.0, 82.56 ± 0.4, 84.21 ± 0.2, and 85.52 ± 0.4 mm and 96.27 ± 2.0, 96.96 ± 1.0, 97.37 ± 3.0, 97.65 ± 1.7, and 97.92 ± 3.1 mm inhibition of ZnO NPs and ascorbic acid [147].Medicinal mushroom *Cordyceps militaris* was used in the synthesis of zinc oxide NPs, and its synthesis was confirmed with a peak at 432.05 cm⁻¹, which corresponds to the Zn-O bond with surface plasmon resonance at 354 nm by Dias et al., These nanoparticles were spherical and irregularly shaped with an average particle size of 1.83 nm, a PDI (Potato Dextrose Agar) value of 0.29, and a zeta potential value of -6.42 mV. Its therapeutic activity through its cytotoxic activity with the brine shrimp lethality bioassay exhibited an Median Lethal Concentration (50%) LC_50_ value of 388.33 ± 66.28; its antidiabetic activity showed an inhibitory effect with α-amylase and α-glucosidase as IC_50_ of 46.29 ± 0.49 and 47.21 ± 0.19 µg/mL; its antioxidant activity using DPPH, ABTS, hydroxyl radical, nitric oxide, and superoxide radical scavenging assays as IC_50_ of 118.63 ± 0.23, 123.43 ± 0.53, 104.52 ± 0.80, and 103.47 ± 1.03 µg/mL, respectively; and its antibacterial activity by agar diffusion assay showed a larger zone for *Pseudomonas aeruginosa* as 22.33 ± 1.52 mm, followed by *Shigella flexneri* − 16.66 ± 1.52 mm, *Proteus vulgaris* − 16.00 ± 1 mm, *Rhodococcus equi-*15.66 ± 1.15 mm, and then Staphylococcus epidermidis − 13.66 ± 1.52 mm. The MIC values were 11.46, 23.40, 11.70, 11.70, and 93.75 µg/mL for *Proteus vulgaris*,* Staphylococcus epidermis*,* Bacillus subtilis*,* Rhodococcus equi*,* Shigella flexneri*, and *Pseudomonas aeruginosa*, whereas with ampicillin it was 0.47, 0.24, 0.03, 0.24, 0.24, and 0.47 µg/mL [148].

#### Fungi-mediated metal oxide nanoparticles

*Zygnema* moss was used to produce copper oxide NPs by the coprecipitation method by Alsalamah et al., and was subjected to High-Performance Liquid Chromatography (HPLC) analysis that used 100 g of *Zygnema moss* powder with 500 ml of methanol and had the presence of 12 compounds: gallic acid, chlorogenic acid, catechin, methyl gallate, caffeic acid, syringic acid, rutin, ellagic acid, coumaric acid, vanillin, ferulic acid, naringenin, rosmarinic acid, daidzein, quercetin, cinnamic acid, kaempferol, and hesperetin. The resultant NPs were spherical in shape, upon on infrared illumination it had with absorption peaks owing to stretching of aromatic alkyl nitro compounds, and metal oxygen, and an absorption peak at 252 nm was observed on UV-visible spectroscopy. Further investigation of its antimicrobial activity showed that *Staphylococcus aureus*,* Escherichia coli*,* Salmonella typhi*,* Enterococcus faecalis*, and *Candida albicans* got 32 ± 0.1, 36 ± 0.1, 27 ± 0.2, 37 ± 0.1, and 34 ± 0.3 mm sized zones of inhibition with 100 µL (100 ppm) of CuO, whereas amoxicillin ( 20 µg/mL) against *Staphylococcus aureus*,* Escherichia coli*,* Salmonella enterica serovar Typhi*, and *Enterococcus faecalis* got 29 ± 0.1, 33 ± 0.2, 25 ± 0.1, and 35 1 mm, and clotrimazole at 1000 µg/mL against Candida albicans got 30 ± 0. The MIC and MBC values concluded by carrying out the microdilution method with spectrophotometric analysis at 620 nm revealed CuO to exhibit 7.8, 7.8, 15.62, 7.8, and 31.25 µg/mL and 15.62, 7.8, 31.25, 7.8, and 31.25 µg/mL against *Staphylococcus aureus*,* Escherichia coli*,* Salmonella enterica serovar Typhi*,* Enterococcus faecalis* and *Candida albicans* [70]. *Cystoseira crinite* brown algae assisted synthesis of magnesium oxide NPs were produced by Fouda et al., by the process extraction got from powder of algae that was obtained after washing, drying and grinding then it was subsequently added with Mg (NO_3_)_2_.6H_2_O with distilled water and incubated for 24 h that gave a colour change from yellow to brownish yellow then it was calcinated at 400֩C and resulted in nanoparticles of MgO to be in size ranging from 21 nm of spherically shaped, with surface plasmons resonance at 320 nm and its antimicrobial activity was monitored through the agar well diffusion method produced zone of inhibition value of 13.3 ± 0.5 mm (50 µg mL^−1^),15.6 ± 0.5 mm (100 µgmL^−1^), 18.6 ± 0.5 mm (200 µg mL^−1^)for Bacillus subtilis, (13.6 ± 0.5 mm) (100 µgmL^−1^) Escherichia coli ,15.3 ± 0.5 mm (200 µg mL^−1^), and 17.3 ± 0.5, 17.6 ± 0.5, and16.6 ± 0.5 mm at 200 µg mL^−1^ for *Staphylococcus aureus*,* Pseudomonas aeruginosa* and *Candida albicans* on the interaction between cell and nanoparticle produced Mg2 + ions that creates alkaline condition unfavourable for pathogen with MIC values 12.5 µg mL^−1^, 50 µg mL^−1^, 25 µg mL^−1^, 50 µg mL^−1^and 12.5 µg mL^−1^ for *Bacillus subtilis*,* Staphylococcus aureus*,* Pseudomonas aeruginosa*,* Escherichia coli* and *Candida albicans* and its In-Vitro Cytotoxicity of MgO with various concentrations (7.8,15.6, 31.25, 62.5, 125, 250, and 500 µg mL^−1^) resulted in 113.4 µg mL^−1^ of IC_50_ concentration against the Caco-2cancer cell line and 141.2 µg mL^−1^ for the normal Vero cell line [149]. The same extract of brown alga *Cystoseira trinodis*, like [150], was used by Gu et al., in the synthesis of CuO NPs by the ultrasound method, obtaining a monoclinic structure and spherical grain-shaped surface morphology with an average crystallite size of 7.43 nm and with O of 19.6 wt% and Cu of 80.4 wt% as its elemental composition. The AFM attributed Rq and Ra values to be 5.27 and 2.96 nm on topographical view and homogeneous grain distribution in 3-D AFM, then with FTIR peaks for CuO stretching and bending at 2384 and 2475 cm⁻¹ and for the bending mode of the CuO bond at 576 cm⁻¹ and for absorbed water at 3400–3500 cm⁻¹, with bands at 298, 336, and 611 cm⁻¹, indicated the presence of three active photons of CuO, with a photoluminescence study demonstrating three peaks at 375, 470, and 535 nm that corresponded to band-edge emission and ionized oxygen vacancy on analyses, whereas on Raman analysis obtained, three active phonons at 298 cm⁻¹ (Ag), 336 cm⁻¹ (Bg), and 611 cm⁻¹ (Bg). Further, its antimicrobial activity by the agar diffusion method against various bacterial strains grown on nutrient agar slants with different concentrations of CuO nanoparticles at concentration 1.25, 2.5, and 5 mg/mL showed 5.36, 8.97 and 16.57 mm for *Enterococcus faecalis* and 4.38, 7.86, 12.62 mm for Salmonella enterica serovar Typhimurium, 7.45, 12.08 and 18.79 mm for Escherichia coli, 4.20, 7.59 and 12.76 mm for Bacillus subtilis, 6.07, 11.53, and 17.48 mm for *Staphylococcus aureus*, 3.57, 6.20 and 13.27 mm *for Enterococcus faecalis*, and found *Escherichia coli* and *Staphylococcus aureus* with the lowest MIC (2.5 µg/mL), whereas the highest MIC was 10.0 µg/mL by *S. typhimurium* with 90 µL of MH broth, 10 µL of bacterial inoculum, and 10 µL of cupric oxide nanoparticles by broth microdilution method with bacteria cultured in MH broth density of 10^8^ CFU/mL, and antioxidant activity by the Brand–Williams method resulted in an IC_50_ value of 543 µg/mL [150]. The research by Kenawy et al., was to synthesize zinc oxide NPs with the alga *Ulva fasciata* by the coprecipitation method, then further add sodium alginate and polyvinyl alcohol for the synthesis of nanofibers by employing the electrospinning technique with four different ratios (PVA: SA) (90:10), (80:20), (70:30), and (60:40) with glycine in different concentrations (0.15, 0.25, 0.5, and 0.75% wt/v). The composite’s antimicrobial characteristic was tested with the agar disc diffusion method, resulting in higher inhibition zones for 10% PVA/1.5% SA/10% CA/0.75% ZnO/0.5% glycine as 32.56 ± 2.06 mm, 30.24 ± 2.04 mm, 28.65 ± 3.21 mm, 21.35 ± 0.98 mm, 16.54 ± 2.33 mm, and 14.21 ± 0.36 mm for *Staphylococcus epidermidis*,* Salmonella paratyphi*,* Escherichia coli*,* Candida albicans*,* Bacillus subtilis*, and *Candida glabrata* [151].

##### Cyanobacterium-mediated metal oxide nanoparticles

Three nanomaterials derived from lichen, namely silver-zinc oxide (Ag–ZnO), silver-strontium oxide (Ag–SrO), and silver-nickel oxide (Ag–NiO) NPs with irregular geometric shapes and were tested on antimicrobial analysis by the agar disk diffusion method against gram-negative bacteria, gram-positive bacteria, and fungus by Kocakaya et al., were with ability of light absorption at 300, 339, and 361 nm on UV analysis with average crystallite size 22 nm, 48 nm and 21 nm, surface charges of -14.9, -23.2, and − 9.64 mV, with PDI values of 0.544, 0.600, and 0.522 of spherical morphology exhibited zones of inhibition for *Escherichia coli* of 6.0 mm, 9.0 mm, 8.5 mm, 8.5 mm, for *Enterobacter aerogenes* as 6.0 mm, 9.0 mm, 8.0 mm 8.5 mm; for *Pseudomonas aeruginosa* as 6.0 mm, 7.0 mm, 8.0 mm, 9.0 mm; for *Proteus mirabilis* as 6.0 mm, 8.0 mm, 9.0 mm, 11.0 mm; for *Enterococcus faecalis* as 6.0 mm, 9.0 mm, 8.5 mm, 10.0 mm; for *Staphylococcus aureus* as 6.0 mm, 10.0 mm, 8.0 mm, 9.0 mm; for *Bacillus subtilis* as 6.0 mm, 9.5 mm, 6.5 mm, 7.0 mm; for *Candida albicans* as 6.0 mm, 19.0 mm, 6.0 mm, 7.0 mm; and for *Candida glabrata* as 6.0 mm, 19.0 mm, 6.0 mm, 8.5 mm for lichen extract assisted Ag–ZnO, Ag–SrO, and Ag–NiO NPs respectively [152].

It is apparent that microbial biomolecules mediated in reducing, nucleation, growth and stabilization in the synthesis and effective surface capping was reflected through enhanced surface charge and colloidal stability [57, 143, 147]. Besides, the progress development of doped and nanocomposite metal oxide system remains under explored. Microbe-assisted synthesis offers advantages such as the production of monodispersed particles with improved crystal structure and chemical composition, and the extracellular mechanism has been found to facilitate a vital process that requires less purification [35, 42, 48]. Among other microbes, bacteria employed in synthesis provide easy culturing and increased growth rates, and we need to investigate how to overcome hurdles in using microbes, such as the difficulty in employing all the strains due to their toxic nature, low yield, agglomeration, and scaling up to large-scale production [10, 31]. The obstacles faced in culture conditions include minor modifications that disrupt metabolic processes, and the costs associated with media and instrumentation for growth must be considered in commercial production, such as low repeatability, expensive machinery, and challenges in obtaining pure nanoparticles [35, 42]. The limitation of using wild-type microorganisms for microbial synthesis is addressed by employing genetically engineered microbes that utilize exogenous genes, enzymes, gene encoding, and the importation of target genes [48, 63].

### Bio-derived materials-assisted synthesis of metal oxide nanoparticles

#### Bio-derived metal oxide nanoparticles

An alternate approach to the biosynthesis synthesis process is utilizing biological derivatives, plant- and animal-based sources, and their byproducts [33, 74]. Copper oxide NPs with spherically clustered scattering on a rough surface were observed on morphological analysis by Banerjee et al., with a value of -3.84 mV as zeta potential and 0.437 as the PDI value. In synthesis with lyophilized powder of cell wall polysaccharides of *Thermotolerant Bacillus anthracis* PFAB2, a star-shaped, flower-like structure and amorphous arrangement were seen in AFM analysis, with a degradation temperature of 283.10 °C and elemental composition of Cu (27.28%) found to be high enough compared to carbon (51.05%), oxygen (33.09%), nitrogen (15.86%), oxygen (20.69%), and sulfur (7.82%). Then the synthesized copper oxide NPs were subjected to antibacterial activity by the standard agar cup method against gram-positive and gram-negative bacteria with various concentrations (1 to 50 g/ml), and the test of their absorbance at 595 nm showed high values on gram-negative bacteria as 33.30 ± 8.30, 41.70 ± 8.30, 66.70 ± 8.30, 58.30 ± 8.30, 125.00 ± 0.00, 100.00 ± 14.40, 116.70 ± 8.30, and 108.30 ± 8.30 mm against *Salmonella typhi*,* Escherichia coli*,* Staphylococcus aureus*, and *Bacillus subtilis* for MIC and MBC [153].Microbial-resistant fabric was woven by Varshney et al., by impregnation of copper oxide NPs over fabric made of cotton. This was observed through Scanning Electron Microscopy (SEM)and exhibited higher activity in the following order against *Staphylococcus aureus* > *Bacillus subtilis* > *Aeromonas species* > *Staphylococcus epidermidis* by electrostatic force that bound the cell and nanoparticle, releasing Cu⁺ ions that generated reactive oxygen species that disintegrated the cell wall and also entering the cellular matrix that destroyed respiratory enzymes of the cell, causing death of the cell, whereas alone CuO, with 78.89% copper and 21.10% oxygen on elemental analysis, polydisperse nature of value 1.0, positive charge of + 4.03 mV on the surface, and 16.23 average crystallite size of spherical shape and broad absorption band at 260–280 nm on UV analysis, at 2.5 mg/ml concentration, the zones of inhibition were 26.66 ± 0.58 mm, 25.66 ± 0.58 mm, and 17.66 ± 0.58 mm for *Staphylococcus aureus*,* Bacillus subtilis*, and *Staphylococcus epidermidis* [154].

Bioderived mesoporous carbon was introduced in the synthesis of iron oxide nanoparticles by Bhosale et al., by the reflux method, and then further characterization uncovered its cubic structure with a 21.44 nm crystallite size on XRD analysis, with bands at 3150 cm⁻¹, 1652 cm⁻¹, 1132 cm⁻¹, 1122 cm⁻¹, 436 cm⁻¹, 533 cm⁻¹, 795 cm⁻¹, and 889 cm⁻¹ on FTIR due to O-H stretching and bending, C-H and C-O groups, and a small granular structure with visible agglomeration on SEM analysis, with a high porous nature of surface area 184.170 m²/g and pore size 13.96 nm on BET analysis and average hydrodynamic diameters of 335 nm on DLS measurement, with a -20 mV value in zeta potential, and its antimicrobial nature was analyzed against 100 µL of *Escherichia coli*,* Pseudomonas aeruginosa*,* Bacillus cereus*, and *Staphylococcus aureus* with nutrient agar as cultures on separate plates and with 10 mg/mL of iron oxide NPs made from mesoporous carbon with dimethyl sulfoxide as a stock solution; the incubation showed effective action through larger inhibition zones that were due to ROS that led to the death of the pathogen through damaging its cell wall, lipids, and proteins [155].

Falemban et al., synthesised NiO NPs first by coprecipitation with Carboxymethyl cellulose (CMC) derived from cellulose as capping agent and with a natural monoterpene D-carvone (Dcar) were all stirred for 3 h was then centrifuged, washed and dried gave NiO-CMC-Dcar then carried out antioxidant test with DPPH radical scavenging assay in which was 1mL of 0.1mM DPPH with methanol as a blank solution with various concentration − 5 µg/mL, 10 µg/ mL, 12.5 µg/mL, 15 µg/mL and 20 µg/mL was checked for colour change after in dark for 30 min absorbance was measured at 517 nm by spectrophotometer resulted in increase in activity with increase in concentration as as 20–100% on using 20 µg/mL is due to surface modification and increased electron transfer, antibacterial activity was carried out by disc diffusion method using muller hinten agar as medium with 100*10^7^ CFU of *Klebsiella pneumoniae*,* Shigella dysenteriae*,* Bacillus subtilis*,* Staphylococcus aureus* and *Candida albicans* gave inhibition zones of 15 mm to 22 mm, and MTT assay for HCT-116 cells was used cultured in Dulbecco’s Modified Eagle Medium with Fetal Bovine Serum, penicillin–streptomycin and L-glutamine with different concentration of synthesised nanoparticle to test apoptosis and cell viability gave IC_50_ value of 10. 2 with cell viability 10% for 20 µg/mL concentration as the synthesised NiO-CMC- Dcar nanocomposite was with crystallite size 24.83 nm and irregular morphology, band gap 4.23 eV, mean particle diameter 130 nm, with elemental composition 8.35% of C indicates the effect of CMC and Dcar component, 49.10% of O and 42.55% of Ni and emission at 386 nm, 426 nm, 455 nm, 482 nm, 506 nm, the oxygen vacancy was at 506 nm that is responsible for ROS production was detected on Photoluminescence (PL) study, diffused ring pattern on SAED and on XPS analysis peaks occurred at 55.26 eV and 860.43 eV, 284.16 eV and 285.88 eV, 528.72 eV, 530.79 eV, and 531.91 eV that corresponds to Ni 2p, O 1 s, and C 1 s [77].Shakerimoghaddam et al., documented the employment of peppermint extract with hydroxyapatite with nickel oxide, and antibacterial activity was observed from previous literature with peppermint extract. This work resulted in spherical-shaped NiO NPs. On testing by the broth microdilution method with blood agar medium that was stabilized with broth and treated at 37 °C, a suspension was prepared, then with 100 µL of sample of 1.5 × 10⁵ CFU/mL concentration and 100 µL of synthesized material for MIC values with concentration (3.25–3.50 mg/mL) as 3.41 ± 0.14 and (1.65–1.75 mg/mL) as 1.7 ± 0.05, and MBC values with concentration (7–14 mg/mL) as 9.33 ± 4 and (3.5–7 mg/mL) as 4.66 ± 2 for *Staphylococcus aureus* and *Pseudomonas aeruginosa*, the biofilm inhibition against 190 µL of 1.5 × 10⁵ CFU/mL for *Pseudomonas aeruginosa* and *Staphylococcus aureus* was 95% and 81%, respectively, for 10 µL of NiO/HAP nanocomposites from a microtiter plate assay [18].

TiO₂ and TiSA NPs were both synthesised with sodium alginate (SA) obtained from brown marine algae by Menon et al., as SA is composed of carboxyl groups and produces cytokines supporting antibacterial properties and also increases oxygen vacancies that increase the ROS production. These are synthesised by dispersing titanium isopropoxide with *Calendula officinalis L.* extract by stirring at 80 °C for 5 h, then the resulting residue was dried at 120 °C for 2 h and calcinated at 300 °C for 5 h to get TiO₂, and by dissolving titanium isopropoxide and sodium alginate in demineralised water with *Calendula officinalis L.* extract by stirring at 80 °C for 4 h, then the solution was dried at 120 °C for 2 h and 300 °C for 5 h to get TiSA nanoparticles. The synthesised exhibited an agglomerated polygonal structure with average crystallite sizes of 32 and 27 nm and a hydrodynamic size of 115 nm, with absorption peaks at 397 nm for TiO₂ and 390 nm for TiSA with bandgaps of 3.12 eV and 3.18 eV, whereas 325 nm as the excitation wavelength proved the presence of oxygen vacancy, and oxygen in the interstitial was proved with peaks at 499 nm and 519 nm, indicating the increased ROS activity with emission peaks at 361, 384, 426, 455, 466, 481, 512, and 532 nm. Both showed effective activity against Gram-positive *Staphylococcus aureus* and *Enterococcus faecium* and Gram-negative *Pseudomonas aeruginosa* and *Acinetobacter baumannii*. and the fungus *Candida albicans* by disrupting cellular processes taking place inside. The particles of TiSA are smaller in size and produced higher band gaps by increasing ROS that damaged the cells, and tissue regeneration of wounds at 10 µg/mL for TiSA was 98 ± 1.09%, whereas for TiO₂ it was 50–55%, and biocompatibility in vitro tests of TiSA produced high cell viability when evaluated by MTT assay [13]. The TiO_2_ NPs with Carrageenan a polysaccharide was test by Majinyari et al., by agar well diffusion method where its antibacterial activity took place by the mechanism of excitation of electron from valence to conduction band leading redox reaction on surface of nanoparticle with production of free radicals that decomposed the pathogen, tested with *Staphylococcus aureus* and *Escherichia coli* grown in lysogenic broth of pH 7 on incubation at 37֩C for 24 h with TiO_2_ dispersed at water in well of petri dish and the further incubated at 37 ֩C for 24 h and zone of inhibition was about 21, 20 mm and 26, 23 mm for carrageenan/TiO_2_ at 450֩C and carrageenan/TiO2 at 650֩C was synthesised by sol gel method and calcinated at 450֩ C and 650֩ C resulted in composite with increased rutile and lessened anatase phase with increase in crystallinity on calcination with average particle radius of 80–160 nm, with surface area from 402.5 to 225.3 m^2^g^− 1^ as the loss functional groups from carrageenan, pore size 13.9 and 18.2 nm and pore volume 0.58 and 0.52 cm^3^g^− 1^ of type IV isotherm of mesoporous nature of closely packed spherical shaped nanocrystals in surface morphology, and the thermal analysis showed improvement in thermal properties owing to the crosslink of polymer with metal particles and weight loss about 15%, 25% and 35% at 100֩C, 249–290֩C, 435֩C was due to water absorbed, sulphur dioxide degradation and carbohydrate backbone fragmentation [156].

Salunke et al., used seaweed *Sargassum cinctum* for the synthesis of zinc oxide NPs, and these NPs produced a single peak with a PdI owing to the value of 0.305 on the DLS study, with a negative value of -13.53 mV on zeta potential, of hexagonal crystal structure with an average size of 29.61 nm on XRD analysis, and rod-shaped particles seen on SEM analysis were further tested for their anticancer activity by MTT assay towards MCF-7 cell lines, and IC_50_ was 98.41 µg/mL by the mechanism of DNA damage, due to the surface area of the nanoparticle and phytoconstituents, polysaccharides and polyphenols present in the extract contributing to anticancerousness [41].A subclass of polyphenols, catechin from green tea, was used in the synthesis of ZnO NPs, which were 20 to 80 nm sized particles with random morphology, by Kader et al., The UV-Vis study exhibited peaks at the UV region for both the extract and ZnO at 329 nm and 278 nm, whereas the nanocomposite peaked at the visible region at 482 nm, with the antimicrobial test’s zone of inhibition being 12 ± 0.57, 9 ± 0.18 mm, 12 ± 0.55, and 10 ± 0.03 mm for *Bacillus cereus*,* Staphylococcus aureus*,* Escherichia coli*, and *Pseudomonas aeruginosa*. Through the Frontier molecular orbitals tool, a decrease in band gap was observed on comparing precursors to the composite formed, and the reason for antibacterial activity was found through molecular docking as the interaction with essential amino acids of *E. coli* after binding with the target [157].The combination of metals has received attention as the studies prove its promising nature through zinc copper oxide NPs (transition metal mixed oxide) were synthesized with resin obtained from the Kerria lacca insect button lac processed as ZnCu(BL)O and raw one-seed lac (ZnCu(SL)O) synthesized by Yadav et al., by the coprecipitation method and characterized and found to be in the size of 31, 22, and 27 nm with rod shape and irregular shape for ZnCuO agglomerated spherical shape for ZnCuBL and ZnCuSL nanoparticles and evaluated the antifungal activity against phytopathogenic fungi using the poisoned food technique with 0.5, 1.0, and 1.5 mg/mL dosages of synthesized nanoparticles against *Alternaria alternata* (0.5 mg/mL) exhibited antifungal activity with ZnCuBL-(O) and ZnCuSL-(O), A (BL), B (SL), and ZnO, and at a lower concentration of 0.5 mg/mL of fungi *Alternaria alternata*, ZnCuBL-(O) and ZnCuSL-(O) nanoparticles exhibited inhibition as 68.21 ± 2.09 and 71.66 ± 4.40 and as 16.85 ± 1.80, 16.95 ± 4.23, and 57.45 ± 2.74 mm for A (BL) and B (SL) and ZnO. These nanoparticles exhibited more activity on *Alternaria alternata* due to their small size, free energy on the surface, and combined effect of both bimetallic NPs, and BL and SL contributed to its antifungal mechanism by ROS (hydroxide and oxide) production owing to crystallographic defects through a mechanism involving six steps. (1) By the endocytosis and diffusion process, enter the cell that produces apoptosis. (2) Membrane disintegration by producing radicals that cause dysfunction by damaging the respiratory chain (3) Release of nanoparticles into the cytoplasm that are insoluble and damage the electron transport system and peptidoglycan (4) Induction of oxidative stress and disintegration of ribosomes through reactive oxygen species and activation of pro-apoptotic proteins (5) Damage of nucleus and DNA (6) Deactivation of enzymes in the cytoplasm with loss of osmotic balance [74].

#### Bio-derived doped metal oxide

A polymer, gum arabic (GA), was used by El-Batal et al., in the synthesis of Ag-CuO NPs by employing a gamma irradiation method that resulted in particles with a face-centered cubic (fcc) crystalline structure with a mean diameter of 25.58 nm and showed its potential activity in various medical areas as an antimicrobial agent against gram-positive bacteria such as *Staphylococcus aureus*,* Bacillus subtilis*, and *Staphylococcus epidermidis*, and gram-negative *Escherichia coli*,* Pseudomonas aeruginosa*,* Acinetobacter calcoaceticus*, and *Klebsiella pneumoniae* in the agar disc diffusion method with µg/mL concentrations of Ag-CuO with zones of inhibition of 20 mm, 22.5 mm, 26.5 mm, 23.5 mm, 20.5 mm, 20.0 mm, and 26 mm, respectively. Ag-CuO NPs, despite the thick membrane of gram-positive pathogens, observed enhanced activity. As an antifungal agent, its activity against *Candida albicans*, the zone of inhibition, was 28.5 mm; as an antibiofilm agent against *Staphylococcus aureus* with 5 mL of nutritious broth with selected bacteria and fungi storing, sodium acetate and deionized water were used to rinse test tubes that were then colored with crystal violet, and discoloration indicated growth of biofilm on all test tubes, showing 88.12%, 87.08%, and 74.0% for *Staphylococcus aureus*,* Candida albicans*, and *Bacillus subtilis* by reducing exopolysaccharide production; as an antiviral agent, there was complete reduction at a concentration of 3.75 µg/mL of Foot-and-Mouth Disease (FDM) virus; and then, in the cytotoxicity test, the cytotoxicity for 25.0 µg/mL was 100%. The clinical test on animals for treating foot-and-mouth diseases caused by viruses A/Africa G4 2022 and A/Venezuela 2022, which are contagious in adult cattle, causing fever, lesions, the formation of painful vesicles, and the development of foot-and-mouth ulcers with nanoscaled Ag-NiO with a dosage of 3.57 µg/mL, showed 100% recovery after 4–5 days and 94% after 7 days for foot lesions, whereas treatment by gentian violet took seven to fifteen days for recovery [28].

#### Bio-derived metal oxide nanocomposites

Chitosan, cerium dioxide, titanium dioxide and polycaprolactone-fabricated CS-CeO₂/TiO₂/PCL and CS-CeO₂ hybrid nanocomposites by Sanmugam et al., were synthesised by sol-gel and got 34 nm and 33 nm as average crystallite size with spindled nanograins, and granulated morphology was subjected to swelling study and degradation with scaffolds of CS-CeO₂ and CS-CeO₂/TiO₂/PCL in buffer solution, revealing their activity due to dual nanoparticles of Ce and Ti with polymer matrix. In an MTT assay for fibroblast (NIH 3T3) and human keratinocyte (HaCaT) cell lines, CS-CeO₂/TiO₂/PCL was found with high cell viability due to increased cell proliferation capabilities by promoting cell migration and adhesion and enhanced cell adhesion by chitosan, and wound healing was tested on albino Wistar rats with continuous monitoring from the zeroth, seventh and fourteenth day, finding 60%, 80% and 93% wound closure by tissue re-epithelialisation, cell proliferation and specifically granulation tissues with control, CS-CeO₂ and CS-CeO₂/TiO₂/PCL scaffolds [158].

PVA (polyvinyl alcohol): SA (sodium alginate): TiO₂ nanocomposite films were prepared in a ratio (PVA: SA 3:1 w/w) by Ibrahim et al. with TiO₂ of different concentrations of 0 wt%, 1 wt%, 3 wt%, and 5 wt% by weight, while employing aloe vera leaves for TiO₂ synthesis by the co-precipitation method carried out by Ibrahim et al., On the increasing TiO_2_ concentration its glass transition temperature increased from 53.7 °C to 60.6 °C similarly its thermal stability and activation energy increased, on zeta potential increase in the negative values conforms the formation and its high stability was then tested on Swelling and degradation studies showed it possessed the ability swelling reduction occurred and water absorption with increasing ratio of TiO_2_ decreased weight loss, antimicrobial analysis against *Bacillus cereus*,* Staphylococcus aureus*,* Candida Albicans* and *Aspergillus niger* was 11.23 ± 0.21 mm, 13.30 ± 0.09 mm, 60.93 ± 0.15 mm and 33.41 ± 0.22 mm, 39.58 ± 0.25 mm, 59.78 ± 0.11 mm, 65.59 ± 0.18 mm and 45.59 ± 0.08 mm, 5.40 ± 0.07 mm, 34.21 ± 0.17 mm, 36.82 ± 0.05 mm and 23.50 ± 0.16 mm, 14.84 ± 0.07 mm, 42.58 ± 0.18 mm, 51.82 ± 0.24 mm and 57.66 ± 0.19 mm for TiO_2_ nanoparticles doped in PVA: SA(wt%)0 wt%, 1 wt%, 3 wt%, and 5 wt% of TiO_2_ and effective activity for Escherichia coli was only for 3 wt%, and 5 wt% of TiO_2_ as 31.66 ± 0.35 mm and 53.25 ± 0.13 mm and cell viability as 97.31, 98.74, 98.18 and 99% for (100 µg/ml) of PVA: SA: TiO₂ nanocomposite using SRB assay on the cytotoxicity test, and its cellular structure was observed with nanocomposite [68].

With Clove oil- Eugenol (Eug) from dried flower buds, was employed in the Hydrothermally synthesis method to get ZnO: CoO / Eug and ZnO: Fe_2_O_3_ / Eug nanocomposite prepared with 0.5 M of Zn(CH_3_CO_2_)_2_ ,0.5 M of Co(CH_3_CO_2_)_2_.4H_2_O / Ferric (III) chloride FeCl_3_ dissolved in deionized water with addition of eugenol and added sodium hydroxide by Al-jubouri et al., that resulted in ZnO: CoO / Eug and ZnO: Fe_2_O_3_ / Eug nanocomposite with stacked sheet and aggregated particles on surface morphology with average crystallite size 59.01 nm and 23.8 nm, and particle size 80 to 90 nm and 60 to 80 nm, with average roughness (Ra) was 4.39 nm and 6.95 nm and the mean standard deviation (Rq) was 6.72 nm and 9.48 nm on AFM analysis, antimicrobial activity with 0.06 gm of nanocomposite dissolved in dimethyl sulfoxide exhibited zone of inhibition of 24, 2, 37 mm for ZnO: CoO and 16, 20, 22 mm with ZnO: Fe_2_O_3_ for *Escherichia Coli*,* Staphylococcus aureus* and *Candida albicans* respectively and the antioxidant activity was observed by dissolving 4 mg of DPPH in deionised water with various ppm 6.25, 12.5, 25, 50 ,100 µg\ml showed IC_50_ values of 26.5, 34.7 and 1.4 for ZnO: CoO / Eug, ZnO: Fe_2_O_3_ / Eug nanocomposite and ascorbic acid [72]. In the coprecipitation method, the biopolymer chitosan was employed to synthesize the Fe₂O₃/ZnO nanocomposite by Al-Rajhi et al., who observed a 20–95 nm size on TEM and prominent peaks at 3462.37 cm⁻¹, 1639.56 cm⁻¹, 1553.73 cm⁻¹, 1412.92 cm⁻¹, 1111.05 cm⁻¹, 1021.35 cm⁻¹, 400–500 cm⁻¹, and 600–900 cm⁻¹ areas, which were attributed to the stretching vibration of –OH, stretching the primary amide’s C–O, bending of the secondary amide group’s N–H, C–N axial shift, C–O–C glycosidic ZnO bond, and the Fe–O group, resulting in potential activity against *Staphylococcus aureus*,* Bacillus subtilis*,* Escherichia coli*, and *Candida albicans* with zones of inhibition of 25 ± 0.1, 28 ± 0.2, 27 ± 0.1, and 27 ± 0.2 mm, whereas there was no activity against *Aspergillus niger* with MIC values of 31.25 ± 1.33 and 15.62 mg/mL. ±0.5, 62.5 ± 0.66, 15.62 ± 1.2, and 15.62 ± 0.33, and MBC 6.25 ± 1.2, 15.62 ± 0.5, 62.5 ± 0.66, 15.62 ± 1.2, and 15.62 ± 0.33, and also exhibited effective antioxidant activity with IC_50_ phytochemical composition and synthesis conditions values of 16.06 and 32.6 µg/mL using DPPH and ABTS scavenging. The wound-healing activity of synthesized nanocomposite at 24 h was 100% wound closure compared to untreated, which was only 76.75%, and biofilm inhibition with 25%, 50%, and 75% of nanocomposite was 66.80%, 74.38%, and 88.60%; 84.88%, 92.21%, and 96.99%; 81.64%, 90.52%, and 94.64%; 85.0%, 93.2%, and 96.0%; and 90.11%, 94.43%, and 98.24% for *Candida albicans*,* Bacillus subtilis*,* Staphylococcus aureus*,* Escherichia coli*, and *Pseudomonas aeruginosa* [159,153].

Incorporation of biological derivatives with metal oxide nanoparticles is versatile and sustainable approach. Metal oxides assisted with biological derivatives formed composite systems that outperformed owing to oxygen vacancies, reduced particle size, ROS and metal ion generation [13, 77]. The polymer addition is not only as a stabilizer but also are functional modifiers [77, 158, 159].

## Analysis and discussion

The prosecution of nanotechnology in biomedical sciences has created an imperative role and wonders in enhancing the effectiveness of materials [25, 50, 160, 161]. Moreover, the adoption of green pathways in nanomaterial synthesis using secondary metabolites provides multiple roles, including reduction, capping, and stabilization, while also employing the principles of green chemistry to enhance safety [10, 19, 32, 36, 68, 112]. This review demonstrates that biosynthesized metal oxide, doped metal oxide, and their nanocomposite nanoparticles show considerable latent application in the therapeutic field as antimicrobial agents. These nanoparticles serve as an alternative to antibiotics because they offer increased biocompatibility with minimal adverse effects, addressing gaps that conventional techniques do not cover in the development of antimicrobial agents assisted by biomolecules [32, 55, 59]. Concurrently, the extract’s biometabolites engineer nanoparticle physicochemical characteristics that bring about a decisive role in antimicrobial efficacy [7, 55, 59].

The biomolecule-assisted synthesis of metal oxide nanoparticles has proven not only biocompatible but also reliable, innocuous products with high stability, offering superior mechanical and thermal stability, enhanced action against microbes, ease of synthesis, and better adsorption and solubility [1, 3, 13, 18, 19, 24, 27, 31–33, 36, 37, 54, 55, 60, 68, 155]. The biomolecule-assisted nanoparticles synthesized by the one-pot method involve synthesis without waste and are free of solvents, but this technique produces non-uniform biomass that’s overcome by the use of an ionic solvent [10]. A comparative assessment with green approaches indicated plant extract-assisted nanoparticles stand out among others by enabling stability, which is the important factor necessary for biomedical applications [10], and affordability on a mass scale of production; acceptable waste products; less time for fabrication; less hazardous, simple disposal; and moderate pH [23, 30, 32, 36, 60, 112].

**Fig. 4:**
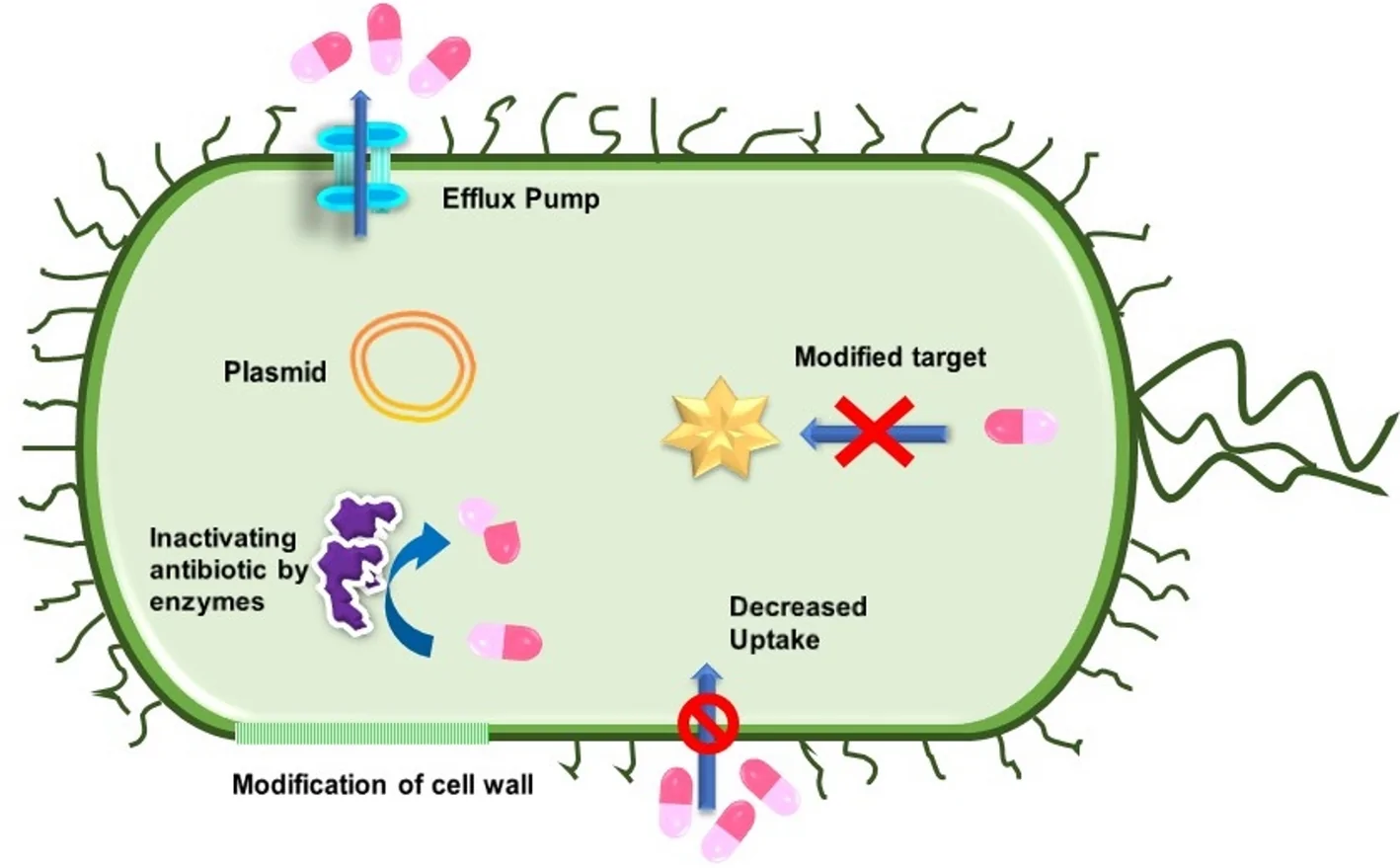
Overview of antibiotic resistance mechanism.

The critical insight of this review is that its superior antimicrobial reactivity largely relies on its small-sized nanoparticles that lead to an increased surface area to act with pathogens and also increased penetration [3, 13, 74, 162]. A wide variety of morphologies were observed, in the implementation of different extracts and biomolecules during synthesis, and effective antimicrobial action requires a morphology that facilitates greater interaction. broadly explored for antimicrobial experiment was ZnO, and also reported to have the smallest crystallite size with the assistance of both plant and microbial extracts [24, 50,131 ,151 ,159 ]. Additionally, metal oxide not only reported on the common antibacterial and antifungal activity but also demonstrated other biofunctions, including antioxidant, antidiabetic, anticorrosive, antiviral, wound healing, and cytotoxic activities; immune regulation; and even notable efficacy against biofilm formation [1, 28, 37, 49, 75, 143, 150, 154]. The antimicrobial performance was identified to be shaped by interconnected factors such as their size, shape, composition, pore volume, surface charge, nature of surface area, band gap, calcination temperature, type of solvent, choice of biomolecule and its parts and its concentration, varying concentration of precursors, and agglomeration tendencies rather than a single attribute [2, 3, 7, 8, 13, 14, 16–18, 21, 23, 25, 38, 51, 56, 74, 76, 77,140 ,156 ]. The nanoparticles’ action against pathogens is either by absorption of these into the microbe surface alone, existing on the surface and creating pores and pits, or by complete penetration [93–98]. The most predominant nanoparticles’ mechanism against microbes is either the absorption of these into the microbe surface alone, existing on the surface and creating pores and pits, or complete penetration that consequently produces membrane disruption, ion release, ROS generation that causes ATP inhibition, and disruption of cellular proteins and DNA [95–98]. The antibiotic resistance mechanism deduced for bacteria was biofilm formation, modifying the target, synthesis of enzymes that modify antibiotics chemically or degrade them, use of efflux pumps to pump out drugs, modifying of drug uptake routes, reducing porin numbers, gene transfer, and cell wall modification. For fungi, it was by modifying the cell membrane through ergosterol reduction, alteration, or replacement; altering the drug binding site through genetic mutations; efflux pump expression; and creation of physical barriers (biofilms). Figure 4 Mechanism of antibiotic resistance [162 –166]. Therapeutic challenge in clinical management is multidrug-resistant pathogens where they exhibit resistance to commonly prescribed antibiotics. Table 2. List out common pathogens with antibiotics, which they developed resistance.

**Table 2:** Major Antimicrobial-Resistant Pathogens and their Common Resistant Antibiotics

Pathogen	Common Resistant Antibiotics
*Staphylococcus aureus*	Methicillin, Oxacillin, Nafcillin, flucloxacillin
*Enterococcus* *faecium*	Vancomycin, Daptomycin, Linezolid
*Klebsiella pneumoniae*	Carbapenems, third generation cephalosporins
*Escherichia coli*	Third generation cephalosporins
*Acinetobacter baumannii*	Colistin, Aminoglycoside,

 [167–169]. These biofilms are dominant factors in producing chronic infections [170]. Biofilms are an extracellular polymeric matrix that is produced by the microbe itself and is made up of polysaccharides, proteins, and eDNA [150,162 ,165].

**Fig. 5:**
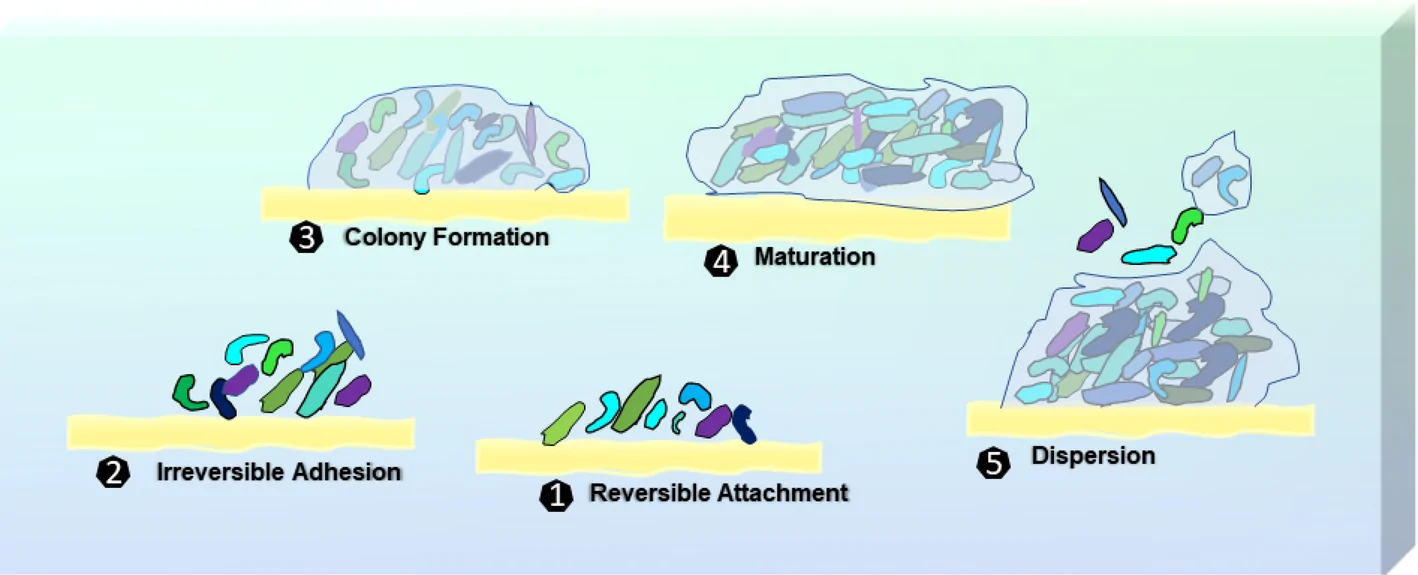
Mechanism of biofilm-mediated antimicrobial resistance

The biofilm’s resistance is through the following steps: initially reversible attachment, then irreversible adhesion for gene expression, colony formation, quorum sensing on maturation, nutrient depletion production, and dispersion. Figure 5 Antimicrobial resistance of biofilms [150,163 , 164]. The nanoparticles’ action against biofilm is by inhibiting autoinducers and degrading the EPS (Extracellular Polymeric Substance) matrix [150, 163]. The bacterial biofilm suppression can be advanced further with magnetic hyperthermia, which uses heat generated by magnetic nanoparticles under an alternating magnetic field; antibacterial photodynamic therapy (APDT), which uses ROS generation with photosensitizer-loaded nanoparticles; antibacterial photothermal therapy (APTT), where heat is used from absorbed light; combination therapy that uses APDT–APTT; and dual-modal nanoparticles with magnetic nature and either APDT or APTT [163]. Furthermore, alterations in the physical properties of nanoparticles directly influence their strength of antimicrobial activity [42]. Refer to Table 3: Action of biomolecule metal oxide nanoparticles against biofilm formation. So, we conclude that the synergistic combination of precursor composition, synthesis parameters, physicochemical characteristics, and optimization is suitable for designing better antimicrobial performance.

**Table 3:** Comparative analysis of Biomolecule assisted metal oxide nanoparticles on biofilm inhibition

Metal oxide NPs	Biomolecule Source	Particle size and Shape	Target microorganism	Concentration	Growth inhibition	Reference
Ag_2_O NPs	*Mauritia flexuos*	110 nm Quasi spherical	*Staphylococcus aureus* *Pseudomonas aeruginosa* *Burkholderia cepacia*	20 µg/mL	71% ± 5 75% ± 16 87% ± 12	[171]
Bi_2_O_3_ NPs	*Curvularia sp*. AS21 ON076460 melanin pigment	19.55nm -	*Citrobacter freundii Enterobacter aerogenes*	200 μL	81.05% 88.25%	[172]
Co_3_O_4_ NPs	Citrus lemon leaf extract	32.26 nm Sieve-like shape	*Escherichia coli* *Staphylococcus aureus*	100 µl	0.096 ± 0.005 mg/ml 0.08 ± 0.007 mg/ml	[﻿^173^]
Mg doped CeO_2_ NPs	*Actinidia deliciosa* peel extract	5 nm Irregular morphology	*Staphylococcus* *aureus*	25 mgmL − 1 50 mg mL − 1 100 mg m	60% 80% 85%	[174]
Cr_2_O_3_ NPs	*Phyllanthus emblica*	large irregularly shaped flakes	*Escherichia coli* *Salmonella enterica*	5 µg/ml	63% 77%	[175]
CuO NPs	Thyme extract	8.98 nm Semi-spherical and irregular	*Klebsiella pneumoniae*	30 mg ml^− 1^	100%	[176]
CuO NPs	*Ficus pumila*	133 nm Cubic to slightly spherical morphology	*Escherichia coli* *Staphylococcus aureus* *Pseudomonas aeruginosa*	100 µg/mL	71 ± 0.12% 62.2 ± 0.02% 67.9 ± 0.03%	[177]
CuO-NPs	*Mentha spicata* leaf extract	17–28 nm Sphere-like form	*Streptococcus mutans*	0.0625 mg/mL	64.15 ± 0.7%	[178]
CuO NPs	*Ononis natrix (L.)*	27.6 nm Spherical	*Marinomonas species* *Staphylococcus aureus* *Psychrobacter species* *Staphylococcus pasteuri* *Bacillus cereus* *Pseudomonas stutzeri* *Enterobacter hormaechei*	1 mL	24.01 ± 22.91% 68.45 ± 12.30% 59.78 ± 16.91 74.19 ± 3.11 86.70 ± 4.92 43.27 ± 19.76 84.66 ± 3.91 72.11 ± 10.69	[179]
CuO NPs	*Opuntia ficus* *indica* leaves	65 nm Spherical	*Pseudomonas aeruginosa* *Methicillin-resistant Staphylococcus aureus*	3.12 µg/mL	8.3% 25.4%	[180]
Cu_2_O: ZnO Nanocomposites	*Curcuma longa*	23.46 nm Uniformly spherical	*Staphylococcus aureus* *Pseudomonas aeruginosa* *Klebsiella pneumoniae* *Enterococcus faecalis*	100 µL	77.98% 75.13% 73.77% 78.15%	[181]
Iron oxide NPs	*Escherichia coli* EPS mediated	< 5 nm Spherical	*Escherichia coli* *Bacillus subtilis*	0.8, and 0.325 µg mL-1 1.05, and 0.8 µg mL-1	69.21% 48.55%	[182]
Iron oxide NPs	*Bacillus subtilis*	< 2 nm Spherical	*Escherichia coli* *Bacillus subtilis*	0.8, and 0.325 µg mL-1 1.05, and 0.8 µg mL-1	72.43% 46.97%	[182]
FeO NPs	*Bauhinia variegata L.*,	< 100 nm Spherical or roughly spherical morphology	*Enterococcus faecium* *Acinetobacter baumannii* *Klebsiella pneumoniae* *Escherichia coli* *Pseudomonas aeruginosa*	1 × MIC	71.78% 73.8% 70.7% 79.1% 75.87%	[183]
FeO NPs	*Avicennia marina*	10–25 nm Spherical	*Pseudomonas aeruginosa* *Escherichia coli* *Staphylococcus aureus*	200 µg/ml	84% 79% 68%	[184]
Fe_2_O_3_ NPs	*Aerva lanta* leaf extract	26 nm spherical shape with light agglomeration	*Bacillus subtilis* *Staphylococcus aureus* *Klebsiella pneumoniae* *Escherichia coli*	100 µg/mL	Approximately 90% for all	[185]
MgO NPs	Orange peel extract	43.57 nm Quasi-spherical shape	*Staphylococcus aureus* *Escherichia coli*	200 µg/mL, 100 µg/mL 50 µg/mL 25 µg/mL 12.5 µg/mL 6.25 µg/mL 3.12 µg/mL	69.6% 66.8% 59.9% 51.6% 46.1% 39.0% 26.7% 55.5% 49.8% 44.7% 39.0% 28.0% 17.7% 10.1%	[186]
NiO-NPs	Eucalyptus globulus leaf extract	19 nm pleomorphism of nanoparticles aggregates	MRSA isolate MSSA isolate *Escherichia coli* *Pseudomonas aeruginosa*	0 mg/ml 0.1 mg/ml 0.2 mg/ml 0.4 mg/ml 0.8 mg/ml 1.6 mg/ml	32% 62% 72% 73% 76% 83% 36% 48% 58% 71% 69% 75% 1% 4% 27% 23% 42% 60% 4% 34% 15% 53% 56% 70%	[187]
TiO₂ NPs	*Cocosnucifera*	17.4 nm Spherical shape	*Escherichia coli* *Staphylococcus aureus*	*-*	70.34% 86.57%	[188]
TiO_2_-NPs	*Linum usitatissimum* seed extract	8.024 nm Irregular or spheroidal rough surface	*Escherichia Coli* *Staphylococcus aureus*	4 µg/mL 8 µg/mL 16 µg/mL 16 µg/mL 32 µg/mL 64 µg/mL	15.66 ± 3.88% 27.70 ± 2.28% 49.73 ± 2.76% 21.84 ± 2.43% 53.27 ± 4.33% 79.96 ± 3.73%	[189]
TiO_2_-ZnO NPs	Onion peel extract	76 nm pentagonal morphologies.	*Methicillin-resistant Staphylococcus aureus*	125 µg/mL 7.8 µg/mL	63.1% 24.7%	[190]
ZnO NPs	Karaya gum	22.7 nm Flower like morphology	*Corynebacterium diphtheriae* *Escherichia coli*	25 µg/mL 50 µg/mL 100 µg/mL 200 µg/mL 400 µg/mL	**3%** **1%** **6%** **3%** **14%** **12%** **26%** **21%** **46%** **34%**	[191]
ZnO NPs	*Medicago sativa* *Euphorbia milii Codiaeum variegatum Helianthus annuus*	33.6600 ± 9.11 nm 36.91839 ± 12.79 nm 37.44992 ± 11.05 nm 37.207 ± 26.18 nm	*Streptococcus pyogenes* *Klebsiella pneumoniae* *Escherichia coli*	30 µL	79% 73.67% 52.3% 7.4% 40.2% 52.4% 56.8% 66.1% 66.4% 60.2% 61.2% 62.0%	[192]
ZnO NPs	Starch and D-glucose	24.41 nm Near spherical morphology	*Klebsiella spp.* *Escherichia coli* *Salmonella typhi* *Staphylococcus aureus*	20 mg/mL 30 mg/mL	28% 33% 27% 48% 41% 38% 37% 53%	[193]
ZnO NPs	*Boswellia Serrate*	-	*Staphylococcus* *aureus*	1 mg/mL 2 mg/mL 4 mg/mL 6 mg/ml	0.95 ± 0.11 0.65 ± 0.07 0.529 ± 0.01 0.226 ± 0.04	[194]
ZnO NPs	Fruit extract of *Annona muricata*	50–100 nm. Spherical	*Enterococcus faecalis*	100 µg mL–1	Decreased biofilm formation in E. faecalis bacteria without cell death.	[195]
ZnO NPs	*Stevia rebaudiana*	11.74 nm Uniform distribution of rectangular, spherical, or semi-spherical particles	MFEC-NT-22 MFEC-S-2	250 µg	86.4% 75.3%	[196]
ZnO NPs	*Alpinia officinarum*	20 nm	*Pseudomonas aeruginosa* *methicillin-resistant Staphylococcus aureus (MRSA)* *Candida species*	-	35% 55% 63%	[197]
ZnO NPs	*Arthrospira platensis*	11–20 nm spherical	*Pseudomonas aeruginosa*	100 µg mL − 1	0.194 ± 0.001 to 0.103 ± 0.001	[198]
ZnO NPs	*Clitoria ternatea*	10–20 nm Spherical	*Enterococcus faecalis Porphyromonas gingivalis*	-	92.27 ± 1.22% 95.27 ± 1.28%,	[199]
ZnO NPs	*Orange peels*	80 nm Semi-spherical with highly agglomerated,	*Pseudomonas aeruginosaPB10 and PA19*	500 µg/mL	> 40% and 80%	[200]
ZnO NPs	*Ery- thrina variegata *leaf extract	29–57 nm Hexagonal and rectangular shaped nanoparticles	*Salmonella Typhi* *Staphylococcus aureus* *Bacillus subtilis*	150 µg/mL	Strong inhibition for all	[201]
ZnO NPs	*Echinochloacolona (E.* *colona) plant aqueous *leaf extract	15.8 nm Spherical and hexagonal shaped nanoparticles	*Escherichia coli* *Klebsiella pneumoniae*	10 µL	80–90%	[202]
ZnO-MgO Nanocomposite	*Sarocladium kilien*	35 nm Aggregated layered structure with flake	*Pseudomonas aeruginosa* *Klebsiella pneumoniae* *Acinetobacter baumannii* *Escherichia coli*	64 µg/mL 256 µg/mL 128 µg/mL 64 µg/mL	82.90% 76.92% 66.31% 54.31%	[203]

## Challenges, future perspectives, and conclusion

Metal oxide nanoparticles synthesized with biomolecules have eminent traits and can be directed towards domains that will provide transformative results, such as biochemical sensors, biomedical applications, catalytic activity, electronic devices, wastewater remediation, dye and heavy metal ion detection, cosmetics, bioimaging, agriculture, biomarkers, and the food industry [19, 28, 32, 39, 46, 55, 113, 118, 138, 150, 156, 163, 157–166, 204–206]. Despite the metal oxide nanoparticles’ abundant benefits, their toxic nature the adverse effect on living cells and long-term stability under physiological conditions, epigenetic alteration demands adequate understanding.

The toxicological profile of nanoparticles is contingent upon their multiple physicochemical attributes. Particle size and surface area are indirectly proportional to chemical kinetics. Particle morphology directs nanoparticle–cell interactions; the spherical-shaped particles were observed to be less toxic than nanoparticles with sharp-edged particles that showed effective action. Surface charge on particles produces electrostatic interaction, where cationic nanoparticles through electrostatic interaction produce high cellular absorption and also triggers platelet aggregation. Notably, highly reactive oxygen species bring better action on pathogens, but that also causes oxidative stress and inflammation to healthy cells. In addition to this, crystalline structure, composition, solvent medium for reaction, coating, and surface roughness regulates catalytic action and response with biological systems, so it is necessary to know the underlying toxic nature through studies. The beneficial aspect of using biomolecules in synthesis is that it reduces toxicity and improves biocompatibility by providing natural capping.

Future researchers have been pushed to tackle current issues of biomolecule-assisted nanoparticles through active research to meet up with the commercialization aspect and elucidate the underlying cytotoxic effect by implementing standardized toxicity testing. Furthermore, substantial investigations are indispensable for biodistribution, immune response, chronic toxicity, and pharmacokinetics. The development of a high-quality, standardized database with artificial intelligence on optimization of experimental reaction parameters, purification strategies, and storage stability can accelerate the biomolecule-assisted nanoparticle’s reproductivity on a large scale, as the bioextract’s phytochemical extraction necessitates standardization of qualitative and quantitative components as they vary across biological sources, leading to the first step being to bring long-term batch consistency. The optimization with algorithms in the experimental process in regard to precursor composition, synthesis parameters, physicochemical characteristics with biological performance facilitate large-scale production. Another notable research gap is inadequate experimental validation for antimicrobial mechanisms that are indispensable rather than proposed ones. Most of the investigations reported enhanced efficiency as an antimicrobial agent by altering multiple physicochemical characteristics simultaneously, making it difficult to distinguish the contribution of individual parameters. Hence, researchers should adopt a controlled experimental design that varies only one physicochemical property at a time while keeping all other properties constant for optimization. A study on quantitative structure–activity relationships through controlled experimental designs where it does quantitative correlation of physical and chemical parameters with antimicrobial outcomes. This emphasizes the need for in vivo assessments and ensuring legal compliance to facilitate the transition from laboratory-scale research to clinical and industrial scales.

In upcoming research should aim to develop nanoparticles with multiple targeting actions, such as targeted therapeutic activity with a combination of cationic, amphiphilic, or receptor-specific ligands. Explore stimuli-responsive nanoparticle coatings, where responsive nanoparticle surface coatings can be activated by light, ultrasound, magnetic fields, and surrounding conditions like pH and enzymatic activity. Nanoparticle encapsulation in hydrogels can be leveraged, where hydrogels, a biocompatible polymer network, provide targeted therapy, reduce toxicity of nanoparticles, and allow gradual release of nanoparticles, reducing dosage of nanoparticles. Additionally, we are in need to overcome the other obstacles, like interpretation of the mechanism of synthesis and dose optimization; less availability of raw material depending on climatic conditions that influence harvest, agglomeration, and dispersion; high pH requirements; polydisperse morphology; less concentration and high efficacy action against pathogens; and difficulties in optimization of physicochemical properties, size, and shape to control as nanoparticle synthesis varies biomolecules’ nature and particles with defective surfaces are necessary to manage the by-product as antimicrobial products end up in water irrespective of chemical or biological nature as waste that leads to accumulation in ecosystems. There is an increasing concern about the need to create a data repository for the precise engineering of nanoparticles, which also requires the development of computational simulation techniques to decode the interactions between biological molecules and nanoparticles during their formation.

In summary, the integration of elements with metal oxide nanoparticles, namely doped and composite creation, leads to multifunctional nanomaterials that complement each other’s limitations, leading to revolutionary innovation that meets the demands of safety, quality, and eco-friendliness. It would then play a transformative role in nanomedicine by minimizing the overall environmental footprint and fabricating nanocomposites with biomolecule-assisted synthesis, antibiotics, polymers, peptides, and multiple elements that would serve as a solution for the reduction of nanoparticle toxicity and aggregation. The incorporation of biomolecules in nanomaterial synthesis would be a promising avenue in the future that aligns with the goal SDG 3: Good Health and Well-being, SDG 6: Clean Water and Sanitation and SDG 9: Industry, Innovation and Infrastructure of creating eco-sustainable and multifunctional products.

## Data Availability

No datasets were generated or analysed during the current study.

## Abbreviations

ABTS: 2,2'-Azino-bis(3-ethylbenzothiazoline-6-sulfonic acid)
AFM: Atomic Force Microscopy
BSA: Bovine Serum Albumin
CFU: Colony Forming Unit
DCFH-DA: 2',7'-Dichlorodihydrofluorescein diacetate
DLS: Dynamic Light Scattering
DPPH: 2,2-Diphenyl-1-picrylhydrazyl
EDAX, EDX, EDS: Energy Dispersive X-ray Spectroscopy
ELISA: Enzyme-Linked Immunosorbent Assay
FESEM: Field Emission Scanning Electron Microscopy
FTIR: Fourier Transform Infrared Spectroscopy
GAE: Gallic Acid Equivalent
HPLC: High Performance Liquid Chromatography
IC_50_: Half Maximal Inhibitory Concentration
LC_50_: Lethal Concentration LC_50_
MBC: Minimum Bactericidal Concentration
MIC: Minimum Inhibitory Concentration
MRS broth: de Man, Rogosa and Sharpe Broth
MTT assay: 3-(4,5-Dimethylthiazol-2-yl)-2,5-diphenyl tetrazolium bromide assay
NPs: Nanoparticles
PDA: Potato Dextrose Agar
PDI: Polydispersity Index
PL: Photoluminescence
QE: Quercetin Equivalent
rGO: Reduced Graphene Oxide
ROS: Reactive Oxygen Species
SAED: Selected Area Electron Diffraction
SEM: Scanning Electron Microscopy
SRB method: Sulforhodamine B Assay
TEM: Transmission Electron Microscopy
TG/DTA: Thermogravimetric Analysis / Differential Thermal Analysis
TTC solution: 2,3,5-Triphenyltetrazolium Chloride
UV: Ultraviolet
UV-DRS: Ultraviolet–Visible Diffuse Reflectance Spectroscopy
W-H Plot: Williamson–Hall Plot
XPS: X-ray Photoelectron Spectroscopy
EPS matrix: Extracellular Polymeric Substance
XRD: X-ray Diffraction
